# TRANSPARENT TESTA GLABRA 1 participates in flowering time regulation in *Arabidopsis thaliana*

**DOI:** 10.7717/peerj.8303

**Published:** 2020-01-20

**Authors:** Barbara A.M. Paffendorf, Rawan Qassrawi, Andrea M. Meys, Laura Trimborn, Andrea Schrader

**Affiliations:** Botanical Institute, Department of Biology, University of Cologne, Cologne, Germany; RWTH Aachen University, Institute for Biology I, Aachen, Germany

**Keywords:** TTG1, Flowering time, PRR, Circadian clock, FT, FLC, bHLH92, *Arabidopsis thaliana*

## Abstract

Pleiotropic regulatory factors mediate concerted responses of the plant’s trait network to endogenous and exogenous cues. TRANSPARENT TESTA GLABRA 1 (TTG1) is such a factor that has been predominantly described as a regulator of early developmental traits. Although its closest homologs LIGHT-REGULATED WD1 (LWD1) and LWD2 affect photoperiodic flowering, a role of TTG1 in flowering time regulation has not been reported. Here we reveal that TTG1 is a regulator of flowering time in *Arabidopsis thaliana* and changes transcript levels of different targets within the flowering time regulatory pathway. *TTG1* mutants flower early and TTG1 overexpression lines flower late at long-day conditions. Consistently, TTG1 can suppress the transcript levels of the floral integrators *FLOWERING LOCUS T* and *SUPPRESSOR OF OVEREXPRESSION OF CO1* and can act as an activator of circadian clock components. Moreover, TTG1 might form feedback loops at the protein level. The TTG1 protein interacts with PSEUDO RESPONSE REGULATOR (PRR)s and basic HELIX-LOOP-HELIX 92 (bHLH92) in yeast. *In planta*, the respective pairs exhibit interesting patterns of localization including a recruitment of TTG1 by PRR5 to subnuclear foci. This mechanism proposes additional layers of regulation by TTG1 and might aid to specify the function of bHLH92. Within another branch of the pathway, TTG1 can elevate *FLOWERING LOCUS C* (*FLC*) transcript levels. FLC mediates signals from the vernalization, ambient temperature and autonomous pathway and the circadian clock is pivotal for the plant to synchronize with diurnal cycles of environmental stimuli like light and temperature. Our results suggest an unexpected positioning of TTG1 upstream of *FLC* and upstream of the circadian clock. In this light, this points to an adaptive value of the role of TTG1 in respect to flowering time regulation.

## Introduction

While a species adapts to ranges of abiotic and biotic conditions, the individual plant must cope with its daily local conditions. It achieves this by integrating various signaling pathways and the current status of the plant itself—for example, its developmental stage or the combination and availability of metabolites. Pleiotropic regulators aid in concerted responses and, thereby, regulate a subset of the plant’s trait network. Due to the depth of insights achieved in the past decades of plant molecular biology, its model species *Arabidopsis thaliana* (*A. thaliana*) (Koornneef & Meinke, 2010) is well suited to analyze such pleiotropic regulators.

TRANSPARENT TESTA GLABRA 1 (TTG1) is one such pleiotropic regulator. The gene is expressed in all major organs of *A. thaliana* including the shoot apical meristem (Walker et al., 1999). *TTG1* encodes a WD40 repeat protein (Walker et al., 1999). The integrity of the protein’s WD40 repeats is crucial to its function and its C-terminus is expected to be of high relevance for the protein’s proper folding and domain structure (Zhang & Schrader, 2017). *TTG1* is known as the head of an evolutionarily conserved gene regulatory network that controls five major traits of adaptive value: seed pigmentation (production of proanthocyanidin), accumulation of anthocyanidins (in seedlings), seed coat mucilage production, trichome and root hair patterning (Zhang et al., 2003). Molecular mechanisms underlying the early developmental traits are under investigation since decades in *A. thaliana* and beyond. Already in 1981, the *ttg1* syndrome was described for induced *A. thaliana* mutants characterized by yellow seeds with transparent testa and by the absence of trichomes (glabrous leaves), anthocyanidin accumulation as well as seed mucilage (Koornneef, 1981). Few additional traits like the carbon partitioning between the seed oil, seed pigment and seed mucilage biosynthetic pathways were analyzed in dependence of TTG1 (Chen et al., 2015; Li et al., 2018). However, surprisingly little is known about the role of TTG1 towards late developmental traits.

One of the most important developmental switches in the plant’s life cycle is the transition from the vegetative to the reproductive phase. The appropriate regulation of flowering time is essential for the reproductive success of plants and, therefore, a key determinator of plant fitness. Several genetically identified pathways that are involved in the regulation of flowering time are influenced by environmental (e.g., vernalization, ambient temperature and photoperiod) and endogenous (e.g., autonomous, gibberellin, circadian clock, age, sugar budget) signals (Blumel, Dally & Jung, 2015). These interwoven regulatory mechanisms converge to the floral integrators *FLOWERING LOCUS T* (*FT*), *SUPPRESSOR OF OVEREXPRESSION OF CO1* (*SOC1*) and also *LEAFY* (*LFY*) (Simpson & Dean, 2002).

CONSTANS (CO) and FT form an important module of the photoperiodic pathway. *CO* expression rises about 8 h after dawn with a peak at night (Suarez-Lopez et al., 2001). The accumulating CO protein activates the florigen gene *FT* in leaves (An et al., 2004; Song et al., 2015). In the night, CO is degraded through the COP1/SPA complex (CONSTITUTIVE PHOTOMORPHO-GENESIS 1/SUPPRESSOR OF PHYA-105) (Jang et al., 2008; Laubinger et al., 2006; Liu et al., 2008). Hence, at long days, sufficient FT protein is formed in the leaves and moves to the shoot apical meristem where it induces flowering (Andres & Coupland, 2012). Although *CO* is expressed under short-day (SD) conditions, it cannot sufficiently induce *FT* expression due to the extended night (Valverde et al., 2004). Consequently, mutants of the photoperiod pathway flower late under long-day (LD) conditions and do not deviate in flowering time from the wild type at SD conditions. One such mutant is the *gigantea* (*gi*) mutant. GI is an activator of CO (Sawa et al., 2007). Its protein levels are also regulated by COP1 in presence of EARLY FLOWERING 3 (ELF3) (Yu et al., 2008).

*A. thaliana* is a facultative LD plant. Winter annual accessions flower late and are responsive to vernalization which reduces the FRIGIDA (FRI)-activated transcript levels of the floral repressor *FLOWERING LOCUS C* (*FLC*) through epigenetic modifications at the *FLC* locus (Deng et al., 2018; Hepworth & Dean, 2015). In the rapid-cycling summer annual accessions, either *FRI* is defective, which reduces *FLC* transcript levels, or the *FLC* allele is weak (Michaels et al., 2003). Low levels of FLC induce flowering as FLC is a suppressor of *FT* (Searle et al., 2006). FT activates the downstream transcription factors *LEAFY* (*LFY*) and *APETALA1* (*AP1*) at the shoot apical meristem and thereby causes flowering when an FT threshold is passed (Turck, Fornara & Coupland, 2008).

*SOC1* acts downstream of *FT* and upstream of *LFY*. Similarly to *FT*, it is directly targeted by the floral repressor FLC (Lee & Lee, 2010) which mediates signals from the autonomous and vernalization response pathways. Both pathways act through suppression of *FLC* expression (Simpson & Dean, 2002). SHORT VEGETATIVE PHASE (SVP) is an interaction partner of FLC (Li et al., 2008) and both are mediators of the ambient temperature pathway (Lee et al., 2007; Simpson & Dean, 2002). Ambient temperature adjusts flowering time in a way that cool temperature delays flowering, whereas warm temperature accelerates flowering (Balasubramanian et al., 2006; Blazquez, Ahn & Weigel, 2003). SVP itself also acts as a direct suppressor of *FT* and *SOC1* (Li et al., 2008). Moreover, SVP can activate members of a group of additional *FT* suppressors, the APETALA2 (AP2) domain containing transcription factors TEMPRANILLO (TEM) 1 and TEM2 (RAV transcription factors with AP2/ERF and B3 DNA-binding domain), AP2 and the AP2-like transcription factors SCHLAFMÜTZE (SMZ), SCHNARCHZAPFEN (SNZ), TARGETs OF EARLY ACTIVATION TAGGED (EAT) (TOE) 1, TOE2 and TOE3 (Tao et al., 2012; Yant, Mathieu & Schmid, 2009). These AP2 domain containing factors act directly at the *FT* gene. TOE1 is able to bind to the *FT* promoter close to the CO-binding site (Zhang et al., 2015). SMZ also seems to affect *FT* expression directly, since *FT* was found as a target of SMZ in a ChIP-chip assay (Mathieu et al., 2009). TEM1 and TEM2 act as *FT* repressors by binding to its 5′ UTR. Furthermore, it is suggested that the balance between TEM and CO controls *FT* transcription and thereby is involved in determination of flowering (Castillejo & Pelaz, 2008).

The AP2 domain containing factors are not only connected with the ambient temperature pathway but also with the gibberellin signaling pathway by TEM1 and TEM2 (Osnato et al., 2012). Moreover, TOE1 interacts with the activating region of CO and the LOV domain of FLAVIN-BINDING KELCH REPEAT F-BOX1 (FKF1). This prevents CO from activating *FT* transcription and FKF1 from stabilizing CO (Zhang et al., 2015).

Upstream of CO, the circadian clock influences the flowering time regulatory pathway. Circadian oscillators are the key for a plant to synchronize with the external environmental cues providing an adaptive advantage (Dodd et al., 2005; Michael et al., 2003). The circadian clock and its feedback loops cause in general rhythmic gene expression within and downstream of the clock. A screen analyzing the MYELOBLASTOSIS (MYB), basic HELIX-LOOP-HELIX (bHLH) and basic region/leucine zipper factors in *A. thaliana* found that 20% of these are under the control of the clock (Hanano et al., 2008). In turn, MYB3R2, bHLH69 and bHLH92 were found to alter clock parameters when overexpressed and therefore might position upstream of the clock (Hanano et al., 2008).

The core negative feedback loop of the clock is formed by the MYB-like proteins CIRCADIAN CLOCK ASSOCIATED 1 (CCA1) and LATE ELONGATED HYPOCOTYL (LHY) which are expressed in the morning and the evening-expressed PSEUDO RESPONSE REGULATOR (PRR) PRR1/TIMING OF CAB EXPRESSION 1 (TOC1) (Oakenfull & Davis, 2017). Several additional loops are formed within the central oscillator. *PRR9*, *PRR7*, *PRR5* and *PRR3* peak successively during the day (Matsushika et al., 2000) filling the gap between *CCA1*/*LHY* and *TOC1*. The PRRs act as suppressors of *CCA1* and *LHY* (Nakamichi et al., 2010). Moreover, GI forms a predicted feedback loop with TOC1 (Locke et al., 2006) and the evening complex consisting of ELF4, ELF3 and LUX ARRHYTHMO (LUX) is required to maintaining circadian rhythms through regulating different key clock genes (Huang & Nusinow, 2016).

The PRR proteins have an N-terminal pseudo-receiver domain which is similar to the phospho-accepting receiver of the two-component response regulators but lacks the presumed phosphor-accepting aspartate. At their C-terminus, a CO, CO-like and TOC1 (CCT) motif is shared by the name-giving proteins (Makino et al., 2000; Matsushika et al., 2000; Strayer et al., 2000). PRRs act antagonistically with *LHY/CCA1* on the downstream CO-FT module (Nakamichi et al., 2007). At the protein level, PRRs interact with and stabilize the CO protein enhancing CO-mediated *FT* transcription (Hayama et al., 2017).

Only few transcriptional activators of the circadian clock are known (Shim, Kubota & Imaizumi, 2017). One of these is LIGHT-REGULATED WD1 (LWD1). LWD1 and LWD2 are the closest homologs of TTG1 that regulate photoperiodic flowering (Wu, Wang & Wu, 2008). Double mutants flower early at LD conditions and exhibit increased *FT* transcript levels (Wu, Wang & Wu, 2008). The *LWD* genes are rhythmically expressed in dependence of PRR9 which forms a feed-back loop with LWD1 (Wang et al., 2011). LWD1 can bind to the promoter of *PRR5*, *PRR9* and *PRR1/TOC1*. With TEOSINTE BRANCHED1-CYCLOIDEA-PCF20 (TCP20) and TCP22 it binds to the *CCA1* promoter activating its expression (Wang et al., 2011; Wu et al., 2016). To date, there was no evidence suggesting an involvement of TTG1 in the regulation of the circadian clock and flowering time. A potential involvement of TTG1 in its transcriptional control has not been reported.

Here we reveal that TTG1 can modulate flowering time along with an initial embedding of TTG1 in the flowering time regulatory pathway. Most strikingly, TTG1 can suppress *FT* and *SOC1* transcript levels and increase those of clock components while reducing their amplitude as observed within one day. PRR proteins can interact with TTG1 in yeast and, when co-expressed *in planta*, exhibit interesting subcellular localization patterns of and with TTG1. In the same systems, TTG1 also interacts with and modulates the localization of bHLH92. Flowering time results at LD conditions as well as the integrators’ transcript levels are in line with an increase of the *FLC* transcript level upon TTG1 overexpression. Together, we suggest that TTG1 acts in multilayered processes in flowering time regulation and it might act upstream of FLC and the clock.

## Materials & Methods

### Plant material und growth conditions

The used *A. thaliana* mutants *ttg1-9*, *ttg1-11*, *ttg1-21*, *ttg1-22*, *glabra3* (*gl3*)*-3*, *enhancer of gl3* (*egl3*)*-19114*, *tansparent testa 8* (*tt8*)*-SALK*, *myc1-1*, *cop1-4* (all Col-0), *ttg1-1* (L*er*), *ttg1-10* (Ws) (Table S7) have been described before (Alonso et al., 2003; Appelhagen et al., 2014; Jakoby et al., 2008; Koornneef, 1981; Larkin et al., 1994; Larkin et al., 1999; McNellis et al., 1994; Pesch et al., 2013; Rosso et al., 2003; Walker et al., 1999; Wester et al., 2009). Primers used for genotyping (including dCAPS primers) are listed in Table S8 (Appelhagen et al., 2011; Jaegle et al., 2016; Neff, Turk & Kalishman, 2002; Schrader et al., 2013). The floral dip method (Clough & Bent, 1998) was used to generate overexpression lines in Col-0 and *cop1-4* background. T_1_ plants were BASTA selected and resistant plants were screened for YFP fluorescence using a Leica stereomicroscope (MZ FLIII) (Leica Microsystems, http://www.leica-microsystems.com). This analysis was repeated for plants homozygous for the insert being at least in T_3_ generation and overexpressing YFP-TTG1 (Table S4). Two walk-in plant chambers were used. Detailed conditions at the respective used areas are listed in Table S1. For flowering time experiments, single seeds were placed in parallelly prepared pots with soil and stratified for 7 d at 5 °C before being transferred to the respective growth condition.

Seedlings for qRT-PCR experiments were sterilized with 70% (v/v) ethanol, 2% NaOCl, stratified at 5 °C and grown on Murashige and Skoog medium containing 1% sucrose at “cold” LD conditions (see Table S1). Seedlings for circadian transcript profiles were snap frozen in liquid nitrogen (∼100 mg) on day eight starting at ZT0 in 4 h intervals until ZT20. Samples used comparing transcript levels in the overexpression lines OE01-03 and OE19-21 were similarly harvested at ZT11 and ZT13 as well as parallelly grown seedlings for comparing protein levels in the same lines at ZT11 and ZT10, respectively. Three biological replicates were analyzed for circadian profiles and comparisons of transcript and protein levels among the TTG1 overexpression lines.

### Phenotyping

Flowering time was recorded as the number of post-stratification days until bolting and the total number of leaves at the time point of bolting. Bolting was defined as the time at which the first bud was visible. Two (bHLH overexpression lines’ experiment) or three experiments (TTG1 overexpression lines, all *ttg1* mutant sets) were conducted for each set of analyzed genotypes and for each condition with at least six individual plants per genotype and experiment. See Table S2 for details.

### Constructs

Gateway™ (Invitrogen™, http://www.invitrogen.com) entry clones containing the coding DNA sequence (CDS) for the respective protein were generated using BP reaction with the previously published vectors *TTG1* pAS2.1 and *GL3* pcACT2 (Pesch et al., 2015), *EGL3* pcACT2, *TT8* pAS2.1, *MYC1* pAS2.1 (vectors provided by M Pesch) or PCR products using primers for *TOC1*, *PRR5*, *PRR7*, *PRR9*, *bHLH92*, *LWD1* and *LWD2* CDS are listed in Table S8 and cDNA from Col-0 seedlings as a template or using the vectors 35S::PRR7:CFP and 35S::PRR9:CFP (Hayama et al., 2017) as a template, respectively. The used entry vector in all cases was pDONR207 (Invitrogen). All entry vectors were sequenced. LWDs were amplified from the cDNA using GGGGACAAGTTTGTACAAAAAAGCAGGCTTAATGGGAACGAGCAGCGATCC and GGGGACCACTTTGTACAAGAAAGCTGGGTTTCAAACCCTGAGAATT for LWD1 and GGGGACAAGTTTGTACAAAAAAGCAGGCTTAATGGTTACGAGCAGCGATCA and GGGGACCACTTTGTACAAGAAAGCTGGGTTTCAGACCCGGAGAATC for LWD2. See Table S9 for the TTG1, LWD1 and LWD2 CDS which were also used in the alignment in Fig. S2. To generate the construct used for the overexpression lines OE01-03 in Col-0 and OE19-OE21 in *cop1-4* background, *TTG1* pDONR207 was recombined using Gateway™ LR Clonase™ (Invitrogen™) into pENSG-YFP (N Medina-Escobar, a version for C-terminal fusions was published before Feys et al., 2005). In analogy, the CDS in pDONR207 for *GL3* (OE04-OE06), *EGL3* (OE07-OE09), *TT8* (OE10-OE12) and *MYC1* (OE13-OE15) were recombined into pENSG-YFP and used to generate the overexpression lines numbered as given in brackets and expressing YFP-bHLH fusion proteins driven by the Pro35s.

For tobacco co-localization experiments, *TTG1* pDONR207 and *PRR5* pDONR207 were recombined into pNmR (Schrader et al., 2013) and *PRR5*, *PRR7*, *PRR9*, *TOC1*, *bHLH92* (all in pDONR207) were recombined into pENSG-CFP (N. Medina-Escobar, a version for C-terminal fusions was published before Feys et al., 2005) and for the Y2H experiments, also in pAS2.1-attR and pACT-attR (Clontech, http://www.clontech.com, modified J.F. Uhrig). *LWD1* pDONR207 and *LWD2* pDONR207 were similarly used in combination with pAS2.1-attR and pACT-attR.

In analogy to YFPattB1-pBat-TL-B-p35s and RFP-HAattB1-pBat-TL-B-p35s previously created as negative controls (Schrader et al., 2013) CFPattB1 was amplified from pENSG-CFP with primers ANS393 and ANS235 and recombined in pBat-TL-B-p35s (Schrader et al., 2013) to obtain CFPattB1-pBat-TL-B-p35s.

### qRT-PCR experiments

About 100 mg of seedlings was harvested for each RNA extraction. RNA extractions were done according to the manufacturer’s instructions (RNeasy Plant Mini Kit, Qiagen, http://www.qiagen.com) using a Tissue Lyser (Qiagen) and followed by DNase I (ThermoFisher, https://www.thermofisher.com) treatment. RNA integrity was tested on a gel prior to cDNA synthesis (SuperScript™ III First-Strand Synthesis System, Invitrogen, or the RevertAid First Strand cDNA Synthesis Kit, ThermoFisher) and RNaseH treatment as suggested before (Martel, Grundemann & Schomig, 2002). A PCR using Elongation factor 1-alpha 1 (EF1ALPHA) primers (Kirik et al., 2007) spanning an intron served as a control to ensure that there was no genomic DNA in the cDNA synthesis (Primers in Table S8).

**Figure 1:**
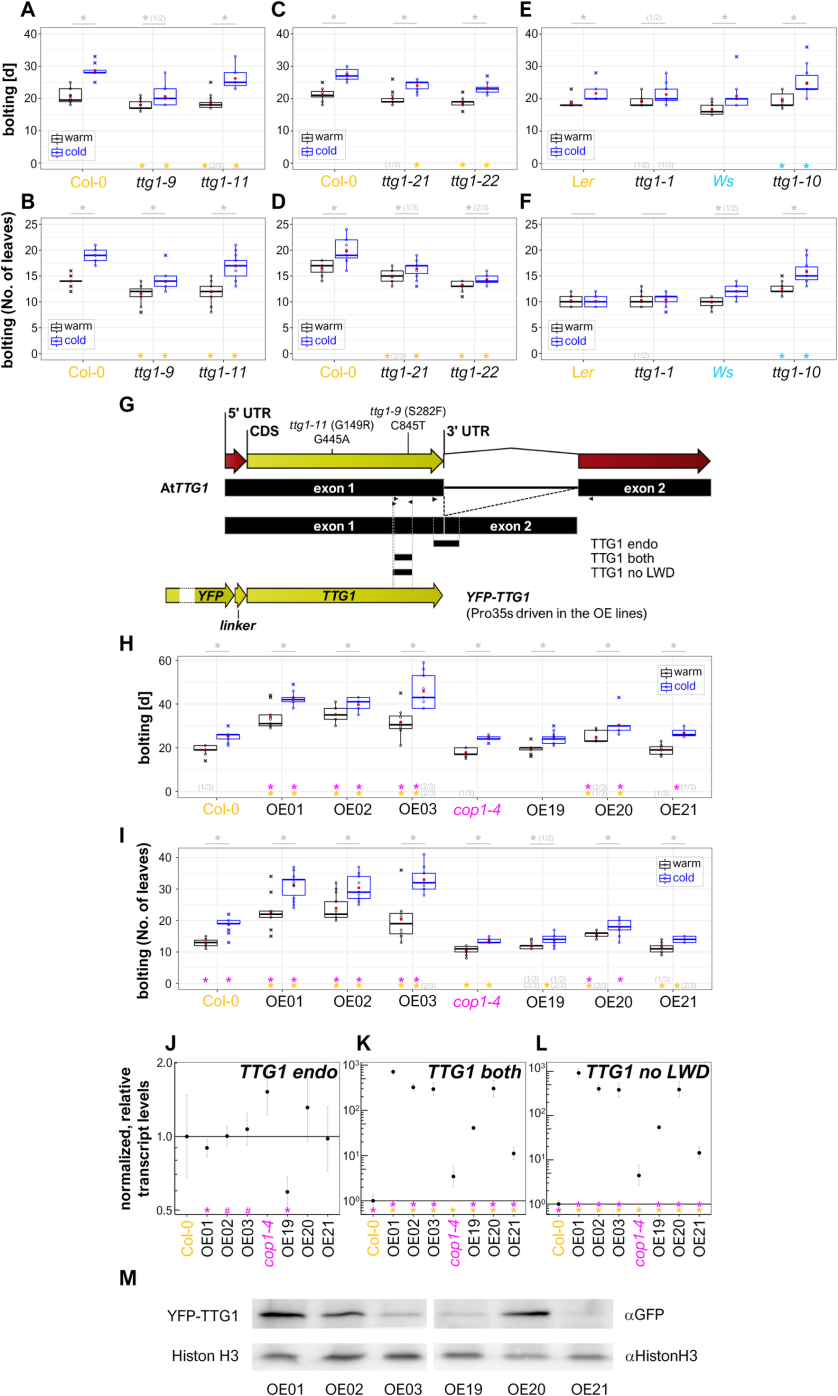
TTG1 has an effect on flowering time regulation in the *A. thaliana* Col-0 ecotype at long-day conditions. (A–D) Flowering time of *ttg1* mutants in Col-0 background. In (A–F, H, I), flowering time was recorded as the number of days until bolting (A, C, E, H) or the number of leaves at the time-point of bolting (B, D, F, I). Plants were grown at long-day conditions (16 h light, 8 h darkness) at 21  °C (“cold”) or 23 °C (“warm”). Black lines in the box plots represent the median, red dots are the mean for the shown representative experiment and crosses mark outliers. Asterisks indicate significant differences (*P < 0.05) between the mutants and the Col-0 wild type (orange) or between the two conditions (grey) in the shown representative experiment. Numbers in brackets indicate the number of experiments for which a significant difference was observed out of the total number of experiments. (G) Primer binding sites for the used primer pairs for qRT-PCR (arrow heads) relative to the *TTG1* genomic, cDNA, CDS and overexpression construct sequence. The position of the point mutations in the *ttg1-9* and *ttg1-11* is indicated above. The T-DNA insertion for *ttg1-21* and *ttg1-22* are provided in Fig. S1 characterizing the mutants’ phenotypes. See Fig. S2 for an alignment of *TTG1* with *LWD1* and *LWD2* in the primer binding region. The sense primers for the primer pairs “TTG both” and “TTG1 no LWD” are overlapping and the sequence of the latter deviates at its 3′ end from the sequence for LWD1 and LWD2. The name “TTG1 both” indicates that the CDS as well as the construct are amplified by this primer pair. (H, I) Flowering time of *TTG1* overexpression lines as described above. (J–M) Characterization of transcript and protein levels in lines overexpressing YFP-*TTG1* in Col-0 (OE01-03) and *cop1-4* (OE19-21) background driven by the 35S promoter. (J–L) Highest levels of *TTG1* transcript were observed in OE01-03 (Col-0) and OE20 (*cop1-4*) while the endogenous *TTG1* transcript levels were in general not affected by overexpression despite in the OE19 (*cop1-4*) line. Note the elevated *TTG1* levels in *cop1-4* mutants. Transcript levels were determined by qRT-PCR relative to *UBQ10* using 8-day-old seedlings and are normalized with Col-0 wild type values. Data represent the mean of three independent experiments. Asterisks indicate significant differences (#, *P* < 0.1; *, *P* < 0.05) between overexpression lines and the backgrounds Col-0 (orange) and *cop1-4* (magenta), respectively. Please note that the scale in (K, L) differs from the scale in (J). The solid line equals 1. The *y*-axis is in log_10_-scaled. Error bars indicate the SD. (M) Western blot using 7d-old long-day grown seedlings form one of the repeats used in (J–L). In OE03 (Col-0), YFP-TTG1 levels varied between experiments never exceeding those in OE01 (Col-0). See also Fig. S3. Tables S2–S4 provide more details on the underlying data and statistics. OE01-03: Pro35s:YFP-*TTG1* (Col-0), three independent insertion lines. OE19-21: Pro35s:YFP-*TTG1* (*cop1-4*), three independent insertion lines.

qRT-PCR was performed using the QuantStudio 5 Real-Time PCR Systems (ThermoFisher) with POWER SYBR Green PCR-Master Mix (Applied Biosystems), the respective cDNA and gene-specific primers. *UBQ10* (*UBIQUITIN10*) was used as a reference gene (Harari-Steinberg, Ohad & Chamovitz, 2001; Sun & Callis, 1997). Three biological replicates with three technical replicates each were performed. Calculations are described in detail in Tables S3 and Table S5. All used primers are listed in Table S8. Most of these were described before (Grigorova et al., 2011; Hayama et al., 2017; Li et al., 2008; Maier et al., 2013; Nakamichi et al., 2007; Shin et al., 2017; Wang et al., 2014; Wang et al., 2011; Wenden et al., 2011; Yu et al., 2012; Zhang et al., 2015; Zou et al., 2013). For TTG1 endo, TTG1 both and TTG1 no LWD see Fig. 1G and Fig. S2.

### Comparison of protein levels

Samples were homogenized under liquid nitrogen to compare YFP-tagged TTG1 in the overexpression lines OE01-03 and OE19-21. 150 µl of lysis buffer (Kirik et al., 2007) were added to the powder and incubated for 30 min at 4 °C (rotating). 100 µl of the supernatant following centrifugation were mixed with 100 µl 2x Laemmli, boiled for 10 min at 95 °C and centrifuged for 1 min at 10 600 g. Samples were separated on SDS-PAGE gels, subsequently blotted and immunodetected (*α*-GFP (IgG1K, Roche), *α*-mouse (Jackson ImmunoResearch, http://www.jacksonimmuno.com)). After GFP detection using the SuperSignal® West Femto Maximum Sensitivity Substrate (ThermoFisher) and a LAS-4000 Mini bioimager (GE Healthcare Life Sciences (formerly Fuji), http://www.gelifesciences.com), blots were stripped as suggested by Abcam (http://www.abcam.com/ps/pdf/protocols/stripping%20for%20reprobing.pdf) using mild stripping buffer (1L: 15 g glycine, 1 g SDS, 10 ml Tween20, pH to 2.2) and re-probed with *α*-histone H3 (ab1791, Abcam, http://www.abcam.com) and *α*-rabbit (A6154, Sigma-Aldrich, http://www.sigmaaldrich.com).

### Y2H experiments and Y2H screening

The TTG1pAS2.1-attR construct (Pesch et al., 2015) was used as bait to screen an *A. thaliana* root cDNA library in yeast (Klopffleisch et al., 2011). Y2H screening was performed as described before using 5mM of 3-AT (Soellick & Uhrig, 2001). Y2H assays were done by co-transformation of pAS2.1-attR/pACT-attR (or TTG1-pcACT2 Pesch et al., 2015) vector combination as described previously (Gietz & Schiestl, 2007). GFPpAS2.1-attR and GFPpACT (Schrader et al., 2013) served as a negative control. At least three replicates were conducted for each Y2H co-transformation experiment and 6 or 8 individual colonies per transformation were resolved in water in 96-well plates and transferred to SD-LW or SD-LWH plates supplemented with different 3-AT concentrations (3, 5, 10, 15, 20, 30 mM) using a 96-well replica plater. Plates were scanned after one (only SD-LW controlling for successful double transformation and providing a relative comparison of transferred yeast amounts), three and seven days.

### Co-localization, microscopy, phenotypic characterization of *ttg1-21* and *ttg1-22*

*Nicothiana benthamiana* leaves were infiltrated as described before (Yang, Li & Qi, 2000) but using the *Agrobacterium tumefaciens* strain GV3101 pMP90RK harboring the respective constructs and Agrobacteria expressing the silencing suppressor TBSV19K (Voinnet, Pinto & Baulcombe, 1999). Infiltrated plants were analyzed three days post-infiltration. CFP-attB1-pBat-TL-B-p35s (this study), YFP-attB1-pBat-TL-B-p35s and RFP-HA-attB1-pBat-TL-B-p35s (Schrader et al., 2013) were used as controls for co-expression with single fluorescent tag fusion protein. The experiment was conducted at least three times for each combination. Infiltrations for the co-expression of CFP and RFP-TTG1 or YFP-TTG1 and RFP, respectively, were included in all experiments. CLSM was performed using a Leica SP8 confocal microscope (Leica Microsystems). Z-stacks were acquired by sequential scanning starting with the laser with the higher wavelength. The LAS Application Suite X (Leica Microsystems) was used to extract and merge images for co-localization figures.

Stacks of small leaves acquired as described before (Failmezger et al., 2013) were merged using Combine ZP (by Alan Hadley, https://combinezp.software.informer.com/) for Fig. S1. Pictures of seeds, seedlings and older leaves (14d-old soil and LD grown) were acquired using a stereo microscope Leica stereomicroscope (MZ FLIII) with the MultiFocus and Montage option of the Leica Application Suite V3 (Leica Microsystems) The step-size was 20 µm (seeds, seedlings) and 50µm (leaves). Seedlings were sterilized as described above and grown on MS (4% sucrose) at constant light at 21 °C.

### Data analysis and statistics

All statistics (Tables S2, S3 and S5), most data analysis, all box plots and plots for qRT-PCR results were generated using R version 3.4.1 (R Core Team, 2017) with the following packages: dplyr (Wickham et al., 2019), extrafont (Chang, 2014), ggplot2 (Wickham, 2016), plyr (Wickham, 2011), scales (Wickham, 2017), tidyr (Wickham & Henry, 2018). Schematics for Fig. 1G, Figs. S1A and S2 were extracted from CLC DNA Workbench (CLC bio A/S, http://www.clcbio.com).

Relative protein amounts were determined using fiji (imageJ 1.52 h, http://imagej.nih.gov/ij). ROIs of the same size for all bands analyzed within both images of one blot detected with both antibody combinations were measured for their mean grey value intensity. The background close to each band was subtracted, GFP values were set relative to the respective Histone H3 values and values obtained for one blot were normalized to OE01 and, in one case for which OE01 was not evaluated, OE20 was used (for OE19-OE21 analysis). Results are shown in Fig. S3.

## Results

### TTG1 has an effect on flowering time

To date, TTG1 has been analyzed in detail for early traits while little is known about its role in the regulation of late developmental traits. When growing *ttg1* mutants, we observed that these flowered slightly earlier than the wild type. Therefore, we selected flowering time as a key late developmental trait. We analyzed the classical *ttg1-9* and *ttg1-11* mutants at the same “warm” long-day conditions (chamber set to 22 °C, on average 23.7 °C at the plant’s level, Table S1). We also analyzed flowering time at an about 2 °C reduced temperature which we named “cold” long-day conditions (chamber set to 20 °C, on average 21.4 °C at the plant’s level, Table S1). Both mutants flowered significantly earlier at both conditions (Figs. 1A–1B, Table S2). *ttg1-9* exhibited the strongest flowering time phenotype and was only slightly responsive to the difference in temperature in contrast to *ttg1-11* and the wild type. Here, the “cold” condition proofed to beneficial in order to reveal differences in flowering time regulation among the *ttg1* mutants.

In both used EMS mutants, an amino acid in the TTG1 protein is changed. These mutants are known to be no null-mutants at least with respect to their effect on trichome patterning. Therefore, we obtained the recently described additional mutants *ttg1-21* and *ttg1-22* in Col-0 background to extend the flowering time analysis (Appelhagen et al., 2014; Rosso et al., 2003). In both mutants, a T-DNA insertion causes a premature stop codon within the inserted T-DNA. The insertion in *ttg1-21* is close to the start and in front of the WD40 domain (Fig. S1A). Therefore, it can be expected that this mutant is a null mutant or at least a comparably strong mutant. In *ttg1-22*, the T-DNA inserts in proximity to the stop codon. This might also causes a strong phenotype as seen for the premature stop codon mutant *ttg1-1* in Landsberg *erecta* (L*er*) background. We tested these mutants for some of the early TTG1-dependent developmental traits. Compared to the wild type, all used Col-0 mutants showed the analyzed aspects of the *ttg1* syndrome (Koornneef, 1981) (Fig. S1). Moreover, we wondered, why the flowering time phenotype of *ttg1* mutants was not reported before. Therefore, we added an often used mutant in L*er* background, *ttg1-1*, a point mutant with a premature stop codon close to the end of TTG1 (Larkin et al., 1994; Walker et al., 1999), and a mutant in Wassilewskija (Ws) background, *ttg1-10,* carrying a point mutation in the *TTG1* promoter (Larkin et al., 1994; Larkin et al., 1999).

The different mutants and variants showed different patterns of flowering time phenotypes relative to the respective backgrounds (Figs. 1C–1F). In Col-0 background, similar to *ttg1-9* and *ttg1-11*, *ttg1-21* and *ttg1-22* flowered significantly earlier in terms of time and leave number at both tested conditions with one exception. For *ttg1-21* grown in the warm condition, the number of days only deviated significantly from the wild type in one out of three repeats. Therefore, early flowering cannot be concluded in this case. For *ttg1-21* and *ttg1-22* it can be summarized that the mutants responded to temperature in the same way as the wild type in regard to time (days) and number of leaves produced until flowering. Again, there was one exception, only in one of three repeats for *ttg1-21*, a significantly different number of leaves was recorded when comparing between the warm and cold condition at flowering time. This suggests that these mutants are less responsive to temperature affecting its number of leaves at flowering time than the wild type and *ttg1-22*.

As compared to the L*er* wild type, flowering time was not significantly reduced in *ttg1-1* at both conditions. Therefore, it is not surprising that the flowering time phenotype was not reported before for this heavily used *ttg1* mutant. In contrast to the other analyzed mutants, *ttg1-10* carries its mutation as a point mutation in the *TTG1* promoter. Interestingly, *ttg1-10* mutants flowered significantly later at both conditions for both recorded flowering phenotypes as compared to its wild type. L*er* and *ttg1-1* did not respond to the difference in temperature, when it comes to the number of leaves produced at flowering time. *ttg1-1* and Ws responded only in one out of two experiments to the difference in temperature for the number of days (*ttg1-1*) and leaves (Ws), respectively. In these cases, a reduced response to temperature cannot be concluded. In all other cases, the reduced temperature caused a delay in flowering time as also observed for Col-0 in the other experiments.

*TTG1-9* and *TTG1-11* encode TTG1 protein variants. Therefore, the observed early flowering might be due to a gain- or loss-of-function of the TTG1 variant or *TTG1* gene in the respective mutants. To specify this, we generated overexpression lines of TTG1 (Col-0) driven by the constitutively active 35s promoter in Col-0 background (Pro35S::YFP-*TTG1* (Col-0), the three lines are subsequently named OE01-OE03, Fig. 1G). As a regulatory hub in light signaling, COP1 is known to regulate protein stability of relevant flowering time regulators (Jang et al., 2008; Liu et al., 2008; Yu et al., 2008) and COP1 can interact with a *TTG1* gene regulatory network component at the protein level (Maier et al., 2013). Therefore, we included *cop1-4* as a background in the flowering time analysis (Pro35S::YFP-*TTG1* (*cop1-4*), the three lines are subsequently named OE19-OE21). All overexpression lines in wild-type background flowered later than the wild type at both temperatures and in respect to time and number of leaves (Figs. 1H–1I). This suggests a loss-of-function in the mutant scenario. Compared to Col-0, *cop1-4* produced significantly less leaves at the time point of flowering. An increase in number of leaves and days in this background was only observed for the overexpression line OE20 as compared to its background (Figs. 1H–1I). This might be due to different transcript or protein levels in the overexpressors.

The overexpression constructs did not affect the endogenous *TTG1* transcript levels despite for OE19 in *cop1-4* background. In this line, a significant reduction of endogenous *TTG1* transcript was observed (Fig. 1J, Table S3, see Fig. 1G and Fig. S2 for the selective primer design). Interestingly, in *cop1-4* mutants, *TTG1* CDS transcript levels were significantly increased (3-4-fold) as compared to Col-0 (Figs. 1J–1L). All overexpression lines showed a significant overexpression of the construct (Figs. 1K–1L). Highest expression and protein levels were reached by line OE01 in Col-0 background and by line OE20 in *cop1-4* background (Figs. 1K–1M, Fig. S3). Both, expression and protein levels, were comparable for line OE02 (Col-0) and line OE20 (*cop1-4*). For OE19 and OE21 (*cop1-4*), in different repeats, YFP-TTG1 levels were either comparable (Fig. 1M) or close to or even below the detection limit (Fig. S3). In Col-0 background, line OE03 exhibited the highest variance of YFP-TTG1 levels compared to other lines in between the repeats. This is in agreement with the observation at the fluorescence stereo microscope using older plants. Here, in several OE03 plants YFP-fluorescence was absent in areas of the leaves or in the center of the rosette. This patchiness of YFP fluorescence was also observed in OE21 (*cop1-4*) and sometimes in OE01 (Col-0) plants but only in one out of 50 OE02 (Col-0) plants and in none of the OE20 (*cop1-4*) plants (Table S4). Therefore, and due to the similar transcript and protein level, OE02 (Col-0) and OE20 (*cop1-4*) were chosen for subsequent quantitative RT-PCR (qRT-PCR) experiments.

Together, we revealed that TTG1 has an effect on flowering time. Subsequently, we used q-RT-PCR experiments for an initial embedding of TTG1 in the transcriptional regulation of the flowering time pathway.

### TTG1 can reduce *FT* and *SOC1* transcript levels

*TTG1* acts early in cell fate determination and is a pleiotropic regulator of transcription. The known mechanisms of TTG1 molecular activity are at the protein level at which it acts in differing complex composition with transcription factors that act as the direct modulators of transcription (e.g., Zhang & Schrader, 2017). Therefore, overexpression lines are most informative to initially reveal if the TTG1 protein can have an impact on the transcriptional regulation of specific targets within the individual branches of the flowering time regulatory pathway. Moreover, on the one hand, by using the *cop1-4* mutant, we added a sensitized background with a significant modulation in protein composition. On the other hand, COP1 interacts with at least one TTG1-complex component (PAP2), therefore, this background also allows for a conclusion if TTG1 activity requires a functional COP1 protein. Results in this background provide insides if TTG1 at elevated protein levels is able to even overwrite the transcriptional scenario in the light-signaling and LD flowering time mutant *cop1-4*. This would underline even more than in the wild-type scenario the potential adaptive value of TTG1 and relevance as a valuable target for flowering time modulation in various environmental settings. All selected targets were analyzed with the most suitable overexpression line as characterized and described above. In addition, seed material of the two EMS mutants *ttg1-9* and *ttg1-11* was available.

First, we assessed the circadian expression profile of endogenous and overexpressed *TTG1* in OE02. As for all circadian qRT-PCR experiments in this study, we used 8-day-old LD grown seedlings harvested first at ZT0 and thereafter in intervals of 4 h with the last sample at ZT20. A similar overexpression level of *TTG1* was seen throughout the day. The endogenous expression was not affected (see Fig. S4, Table S5).

**Figure 2:**
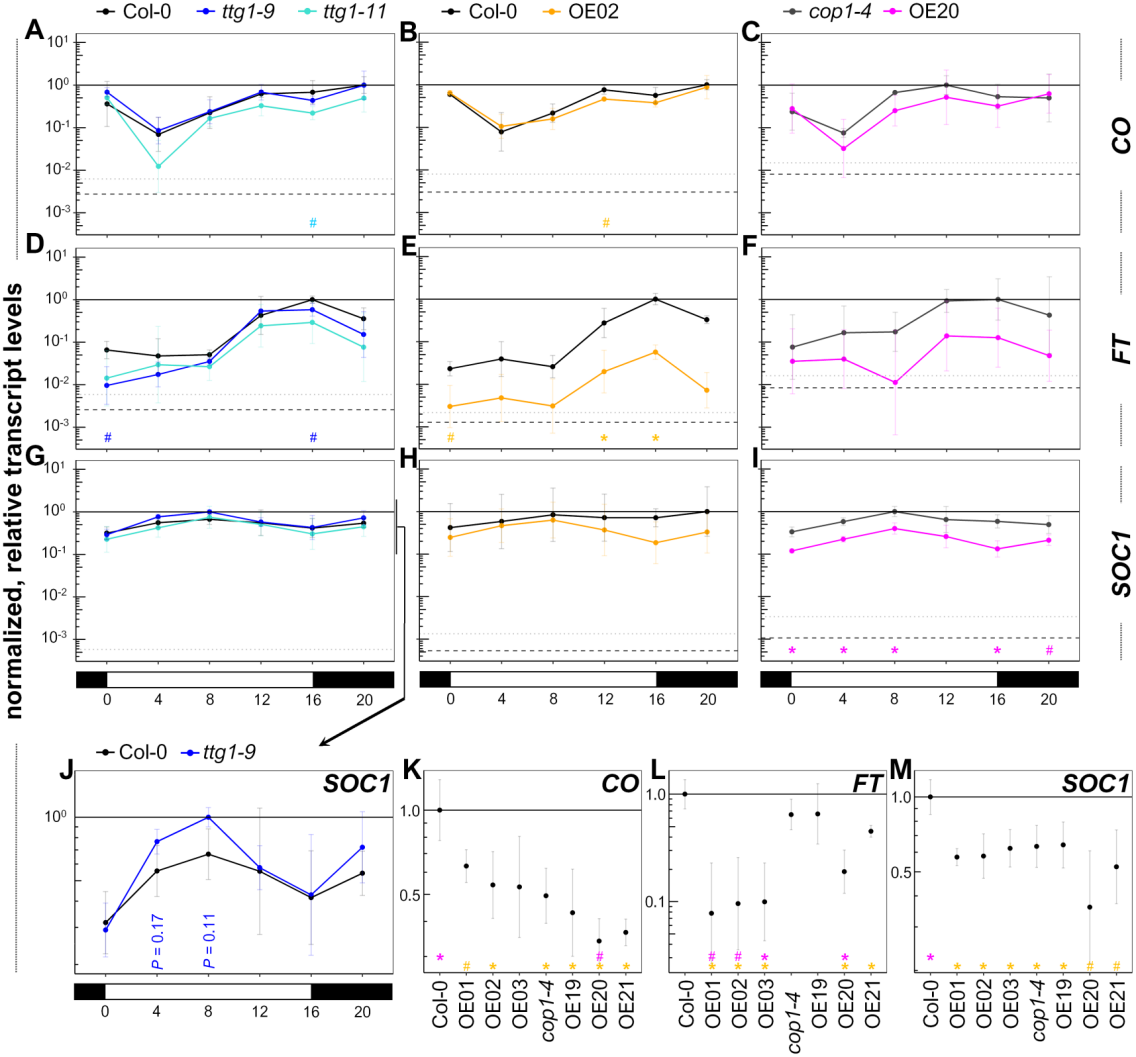
*TTG1* overexpression suppresses *FT* and *SOC1* transcript levels which can not be explained by *CO* transcript levels. (A–J) Eight-day-old seedlings grown under LD conditions (light: ZT0-16, dark: ZT16-0) at 21 °C were harvested in 4h-intervals starting at ZT0. The analyzed genotypes were *ttg1* mutants in Col-0 background, TTG1 overexpression lines in Col-0 (OE02) and in *cop1-4* (OE20) background with their respective backgrounds. *CO*, *FT* and *SOC1* transcript levels were analyzed. Transcript levels are presented relative to the *UBQ10* transcript levels and normalized with the maximal mean per target within a genotype set. (K–M) Same growth conditions as used for A–J. The seedlings were harvested at ZT = 11 and ZT = 13 from OE01-03 (Pro35s:YFP-*TTG1* (Col-0), three independent insertion lines) and OE19-21 (Pro35s:YFP-*TTG1* (*cop1-4*), three independent insertion lines), Col-0 and *cop1-4*. Data are means from three biological replicates originating from three independent seed batches (A–J) or from one seed batch of parallely grown parental plants (K–M). Error bars are SD. Asterisks indicate significant differences (#*P* < 0.1, *P < 0.05) between the mutants (blue: *ttg1-9*, cyan: *ttg1-11*) and their background or the overexpression lines and their respective backgrounds Col-0 (orange) and *cop1-4* (magenta). Dashed line: averaged lower threshold in the set of experiments for the respective target (*Ct* = 35) relative to the respective *UBQ10* levels. Dotted line: average lower threshold + SD. The solid line equals 1. The *y*-axis is log_10_-scaled. See Table S3 for more details on the underlying data and statistics.

Due to the strong late flowering phenotype of the overexpression lines, we expected that the transcript levels of the floral integrators (e.g., Blumel, Dally & Jung, 2015) differed from the respective backgrounds. Towards this end, we continued with analyzing the *CO*-*FT* module’s and *SOC1* transcript levels. In line with the flowering time phenotype, for CO, an activator of *FT* (Suarez-Lopez et al., 2001), a slight tendency to lower transcript levels in the overexpression lines was observed. Nevertheless, this was neither significant nor sufficient to explain the strong phenotype especially in the overexpression in wild-type background (Fig. 2, Table S5). GI is an *FT* regulator that can increase *CO* transcript levels but can also activate FT in a CO-independent way. It can directly bind to the *FT* promoter and interacts with *FT* suppressors (Sawa & Kay, 2011; Sawa et al., 2007). Also, *GI* transcript levels in OE02 were not significantly changed (Fig. S5). In both overexpression lines, for *FT*, the transcript levels almost dropped to the detection limit and exhibited a general reduction at all timepoints which was significant at ZT12 and 16 in OE02. The overexpressors’ *SOC1* transcript profiles were very similar in their circadian pattern and both exhibited reductions throughout the day, which was significant at ZT0, 4, 8 and 16 for OE20. In the mutant scenario, we expected to find more subtle effects. We found only a trend to elevated *SOC1* levels in *ttg1-9* mutants which might explain the early flowering time phenotype but was not significant. In summary, we found that TTG1 can reduce *FT* and *SOC1* transcript levels.

### Early and late effects on transcript levels of AP2-domain containing factors in *ttg1* mutants

**Figure 3:**
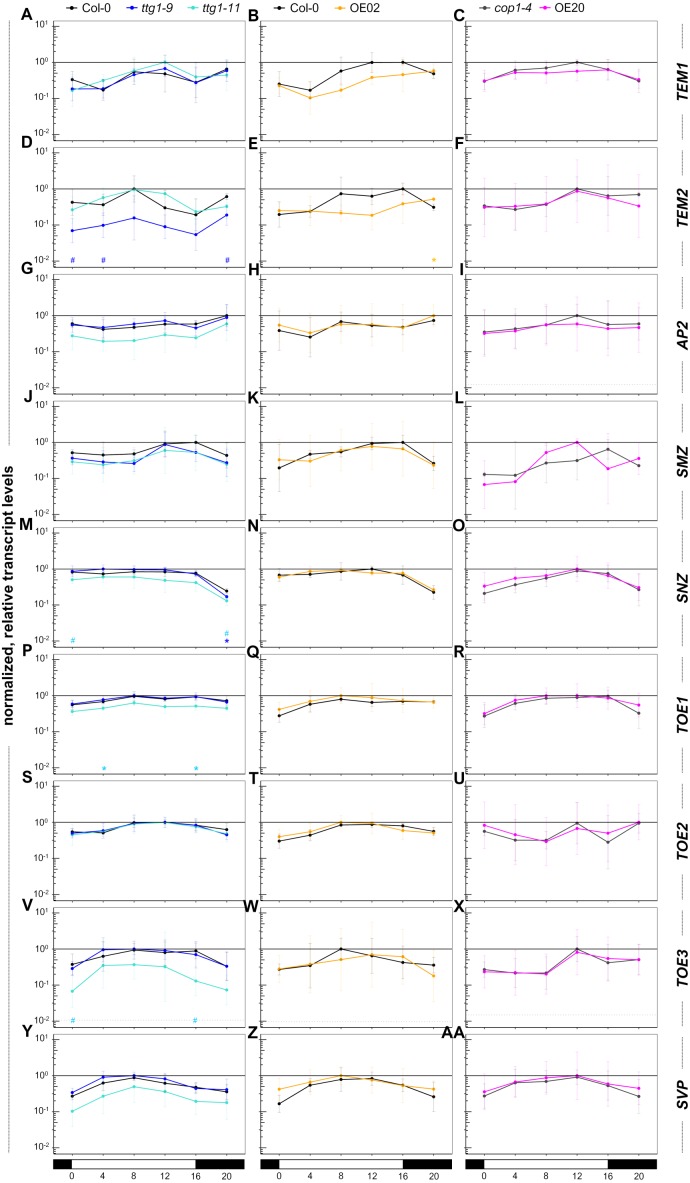
AP2-domain containing factors: reduction of *TEM2*, *SNZ*, *TOE1* and *TOE3* transcript levels in *ttg1* mutants occurs early and late under long-day conditions. In the mutants and overexpressors, we analyzed the transcript levels of transcriptional *FT* suppressors: AP2 family genes *AP2*, *SMZ*, *SNZ*, *TOE1*, *TOE2*, *TOE3,* the two RAV factors *TEM1* and *TEM2* as well as *SVP*, an activator of AP2 family genes and suppressor of *FT*. The same samples as in Fig. 2 were used. Data are means from three biological replicates. Error bars are SD. Dotted line: average lower threshold in the set of experiments for the respective target (*Ct* = 35) relative to the respective *UBQ10* levels + SD. The solid line equals 1. The *y*-axis is in log_10_-scaled. For details on the genotypes and data presentation please refer to Fig. 2 and for details on the underlying data and statistics to Table S5.

We moved our focus on factors that can suppress *FT* transcript levels, the AP2 domain containing factors of the flowering time regulatory pathway: TEM1, TEM2 (RAV transcription factors with AP2/ERF and B3 DNA-binding domain) and AP2, SMZ, SNZ, TOE1-3 e.g., (Song, Ito & Imaizumi, 2013; Wang, 2014). In addition, we tested the transcript level of *SVP* which acts as an activator of the aforementioned AP2-like factors and as a suppressor of *FT* (Lee et al., 2007; Tao et al., 2012). Only at night, a significant increase of the *TEM2* transcript level was observed for OE02 (Fig. 3, Table S5). No significant change was found for OE20 suggesting that elevated TTG1 levels do not have a strong impact on these genes. However, in the mutants’ case a trend for reduced transcript levels was observed for *TEM2* (*ttg1-9*), *AP2*, *TOE1* and *TOE3* (all *ttg1-11*) and a slight reduction for *SNZ* (*ttg1-11*, at night also for *ttg1-9*) and *SMZ* (both mutants). Significance analysis (*P* < 0.05) supported the reduction of *TOE1* transcript levels at ZT4 and ZT16 for *ttg1-11* and *SNZ* transcript levels at ZT20 for *ttg1-9*. Interestingly, although not supported by the significance analysis, the trend of reduced transcript levels for *SVP* seemed to be opposed by elevated *SVP* levels at ZT0 and ZT20 for OE02 pointing to a possibly flattened circadian amplitude of *SVP* transcript levels upon TTG1 overexpression. Therefore, we had a closer look on circadian clock components for OE02.

### TTG1 can regulate circadian clock components

**Figure 4:**
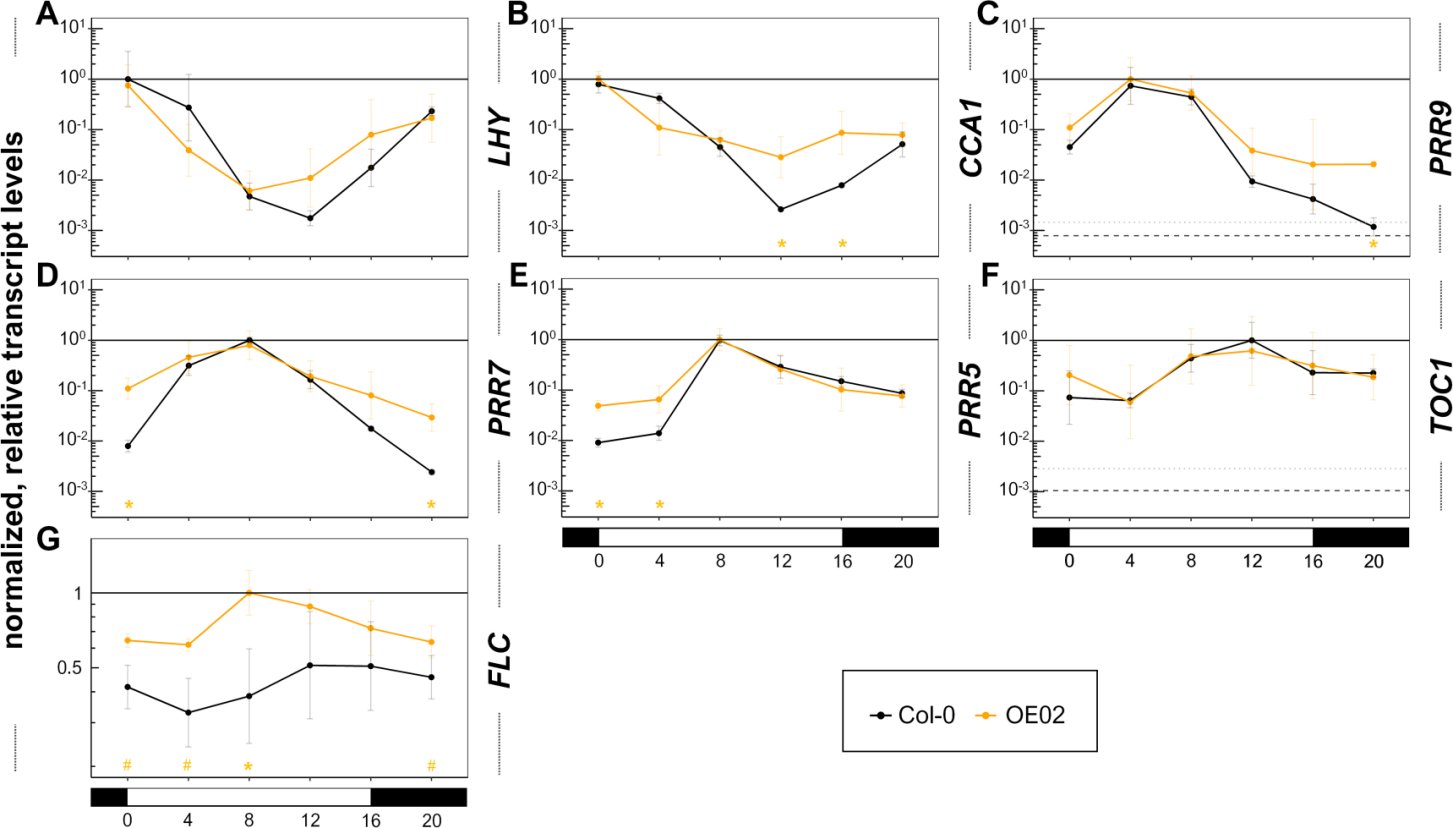
TTG1 can modulate transcript levels of circadian clock components, flattens their circadian amplitude and *TTG1* overexpression increases *FLC* transcript levels. To further explore an additional relevant part of the flowering time regulatory pathway, we used the overexpression line in Col-0 background to analyze eight-day-old seedlings grown under LD conditions (light: ZT0-16, dark: ZT16-0) at 21 °C which were harvested in 4 h-intervals starting at ZT0. (A–F) The analyzed clock components are sorted according to their peak during the day. (G) The selected overexpression line in Col-0 background (OE02) was also employed to further explore another relevant FT-suppressive branch of the flowering time regulatory pathway: FLC transcript levels. All transcript levels are relative to that of *UBQ10* and normalized with the maximal mean per target. Data are means from three biological replicates. Error bars are SD. Asterisks indicate significant differences (*P < 0.05) between the overexpression line and the Col-0 wild type. Dashed line: averaged lower threshold in the set of experiments for the respective target (*Ct* = 35) relative to the respective *UBQ10* levels. Dotted line: average lower threshold + SD. The solid line equals 1. The *y*-axis is in log_10_-scaled. See Table S5 for more details on the underlying data and statistics.

LWD1 and LWD2, the closest homologs of TTG1, regulate flowering through transcriptional modulations within the circadian clock (Wang et al., 2011; Wu et al., 2016; Wu, Wang & Wu, 2008). LWD1 was shown to bind to the promoter of *PRR5*, *PRR9* and *PRR1/TOC1* (Wang et al., 2011). Therefore, we analyzed the transcript levels of the core clock components *LHY*, *CCA1* and *TOC1/PRR1* and also those of its feed-back regulators *PRR5*, *PRR7* and *PRR9* (Shim, Kubota & Imaizumi, 2017) in the TTG1 overexpression line OE02. Figure 4 shows the results sorted by the time point of the maximal peak in the wild type. In general, a flattened circadian amplitude is to be seen despite for *TOC1* which is not affected by TTG1 overexpression. For *LHY*, the minimum seems to be shifted to ZT8 instead of ZT12 in the wild type. A significant increase (*P* < 0.5) in transcript levels was observed for *CCA1* at ZT12 and ZT16, for *PRR9* at ZT20, for *PRR7* at ZT20 and ZT0 and for *PRR5* at ZT0 and ZT4. Thus, we found that TTG1 can regulate the transcript levels of circadian clock components and modulates their transcriptional profiles mainly through flattening the amplitude as a consequence of elevated transcript levels at distinct timepoints on the day under investigation. Similar as its homologs LWD1 and LWD2, TTG1 can change the transcript levels of *PRRs* but did not modulate *TOC1* transcript levels.

The transcript levels of the so far analyzed branches of the flowering time regulatory pathway did not provide a convincing explanation for the strong suppression of *FT* and *SOC1* transcript levels in the overexpression lines. *FLC* represents and integrates additional branches of this pathway. The FLC protein can bind directly to the promoters of *FT* and *SOC1* and suppresses the respective transcript levels (Helliwell et al., 2006; Searle et al., 2006). Therefore, we decided to complete our initial embedding of TTG1 in the transcriptional control of the flowering time pathway by analyzing the transcript levels of *FLC*. We found that overexpression of TTG1 resulted in elevated *FLC* transcript levels throughout the day (Fig. 4). These were significantly and more than 2-fold increased as compared to the wild type at ZT8. We conclude that TTG1 can act as an activator of *FLC*.

One well selected and characterized overexpression line was analyzed in three biological replicates based on three independent seed batches for this initial embedding of TTG1 in the flowering time regulatory pathway. The different and specific time points of transcript modulation spread throughout the day based on the overexpression line OE02 suggest the general ability of TTG1 to elevate transcript levels of clock components and FLC and identify both branches as targets for specific and more detailed follow up studies.

Together, we found that TTG1 acts as a transcriptional regulator in various parts of the flowering time regulatory pathway.

### PRR5 recruits TTG1 to subnuclear foci and bHLH92 nuclear enrichment is counteracted by TTG1

In its role as a regulator of early developmental traits, TTG1 acts through differential complex composition, it acts in competitive scenarios, trapping to the nucleus and mutual localization with respective interactors (Balkunde, Bouyer & Hulskamp, 2011; Bouyer et al., 2008; Pesch et al., 2015; Wester et al., 2009; Zhang et al., 2019; Zhang & Schrader, 2017; Zhao et al., 2008). All these scenarios occur at the protein level in dependence of its interactors. bHLH factors like GL3, EGL3, TT8 or MYC1 and R2R3-MYB factors form R2R3MYB-bHLH-WD40 (MBW) complexes with TTG1 to regulate the early TTG1-dependent traits (Balkunde, Pesch & Hulskamp, 2010; Broun, 2005; Koornneef, 1981; Lepiniec et al., 2006; Miller, Chezem & Clay, 2015; Ramsay & Glover, 2005; Tominaga-Wada, Ishida & Wada, 2011; Walker et al., 1999; Zhang et al., 2003). We wondered, which interactors might be relevant for TTG1 function towards flowering time regulation. As the classical bHLH interactors did not modulate flowering time in a similar way as TTG1 (Fig. S6), we conducted a Y2H screening to identify candidates which are related to the flowering time regulatory pathway. Among the results of this screen was one of the classical bHLH interactors, EGL3 (Table S6) (Zhang et al., 2003). In addition, and related to the flowering time regulatory pathway, we identified PRR5 and bHLH92, a bHLH factor shown to be expressed in a circadian pattern (Hanano et al., 2008).

**Figure 5:**
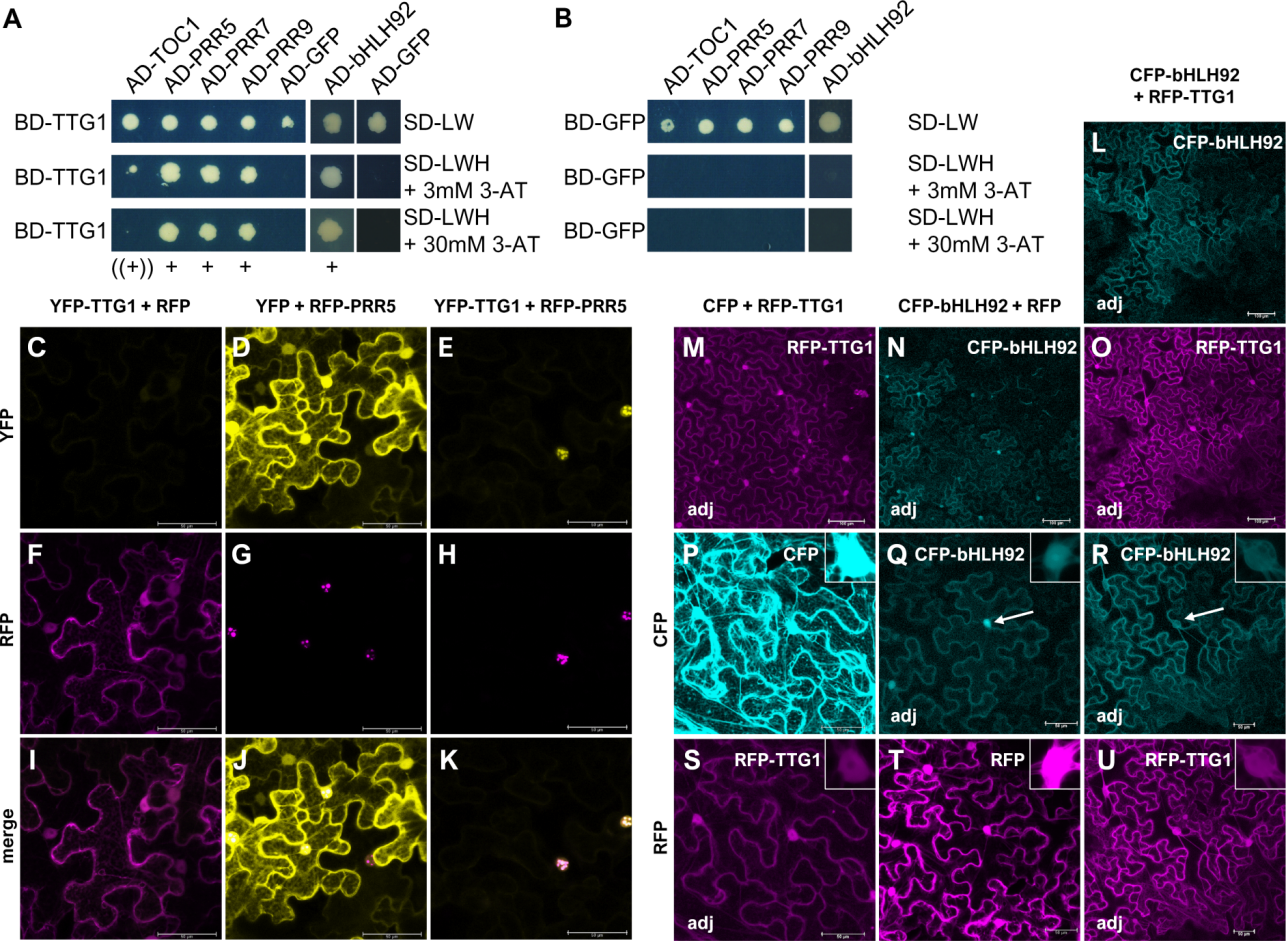
TTG1 interacts with PRR5 and bHLH92 in yeast with suggested functional relevance due to protein re-localizations *in planta*. (A–B) Yeast two-hybrid assay with TTG1 as a bait (A). TTG1 was tested for interaction with the PRRs (TOC1/PRR1, PRR5, PRR7, PRR9) and bHLH92. GFP serves as a negative control (B). Yeast colonies were transferred to interaction plates (SD-LWH) and plates to test for successful co-transformation (SD-LW). Interaction plates were supplemented with different 3-AT concentrations: 3, 5, 10, 15, 20, 30 mM to qualitatively assess differences in interaction strength. See Fig. S7 for additional results discussed in the text. AD = GAL4-activation domain; BD = GAL4-DNA-binding domain (used for bait constructs); SD = synthetic defined medium. (C–U) Representative sequentially scanned, merged confocal stacks of *N. benthamiana* leaf epidermal cells co-expressing RFP- and YFP- (C–K) or CFP- tagged (L–U) proteins. (C-U) YFP-TTG1 is recruited by RFP-PRR5 to subnuclear foci. Constructs were co-infiltrated to co-express YFP-TTG1 and RFP (C, F, I), YFP and RFP-PRR5 (D, G, J) or YFP-TTG1 and RFP-PRR5 (E, H, K) as also indicated above each column. The indicated channels (left of the respective row) were subsequently merged (I–K) and no brightness-contrast correction was applied. Please note that we did neither differentially adjust the detection nor the pictures for YFP among the combinations to improve visualization of the faint YFP-TTG1 signal in order to visualize the strong effect of RFP-PRR5 on YFP-TTG1 protein localization and abundance within the nucleus (E). (L–U) Enrichment of CFP-bHLH92 in the nucleus is reduced when RFP-TTG1 is co-expressed. Constructs were co-infiltrated to co-express CFP and RFP-TTG1 (M, P, S), CFP-bHLH92 and RFP (N, Q, T) and CFP-bHLH92 and RFP-TTG1 (L, O, R, U) as also indicated above each column. L–O show infiltrated leaf areas of the indicated tagged protein in the respective channel. The same post-acquisition brightness and contrast adjustment was applied to all pictures marked with “adj”. White arrows point to representative nuclei with differing, relative CFP-bHL92 enrichment as compared to the cytoplasm and in dependence on the presence of RFP-TTG1. All pictures within C-U were acquired with the same settings for RFP/YFP and RFP/CFP, respectively, despite for a reduced laser intensity for the RFP detection of the CFP-bHLH92/RFP combination (T) and a smaller image size for the CFP/RFP-TTG1 combination (P, S) (512 × 512 as compared to 2,058 × 2,058 px^2^). In Fig. S9 we provide adjusted and non-adjusted pictures for RFP-TTG1 to visualize the protein’s localization. See also Fig. 6 for CFP-PRR/RFP-TTG1 combinations. Bars equal 100 µm in the leaf area pictures and 50 µm in all other pictures. Additional confocal images are shown in Figs. S8–S9. All experiments in the figure were at least conducted three times independently with the same results.

Both selected candidates could be verified as interactors of TTG1 in Y2H experiments in which they were fused to the GAL4-binding as well as to the GAL4-activation domain (Figs. 5A–5B, Figs. S7A–S7B). We also tested PRR7, PRR9 and TOC1 in the GAL4 system (Figs. 5A–5B) with different 3-AT (3-amino-1,2,3triazole) concentrations and an adjusted optical density of the samples (Figs. S7A–S7B). All three PRRs interacted with TTG1 when fused to the activation domain of GAL4. TOC1 exhibited the weakest interaction, as indicated by growth of yeast on the respective plates, followed by PRR9, PRR7 and PRR5. A very weak possible interaction of LWD1 and LWD2 was seen with PRR5 in yeast (Figs. S7B–S7C). However, among the homologs, the interaction with all PRRs is TTG1-specific. bHLH92 did neither interact with LWD1, LWD2 nor with the PRRs in this assay (Figs. S7B–S7C). This suggests that the mechanisms of LWDs and of the TTG1 protein in flowering time regulation including the transcriptional modification of the circadian clock differ.

**Figure 6:**
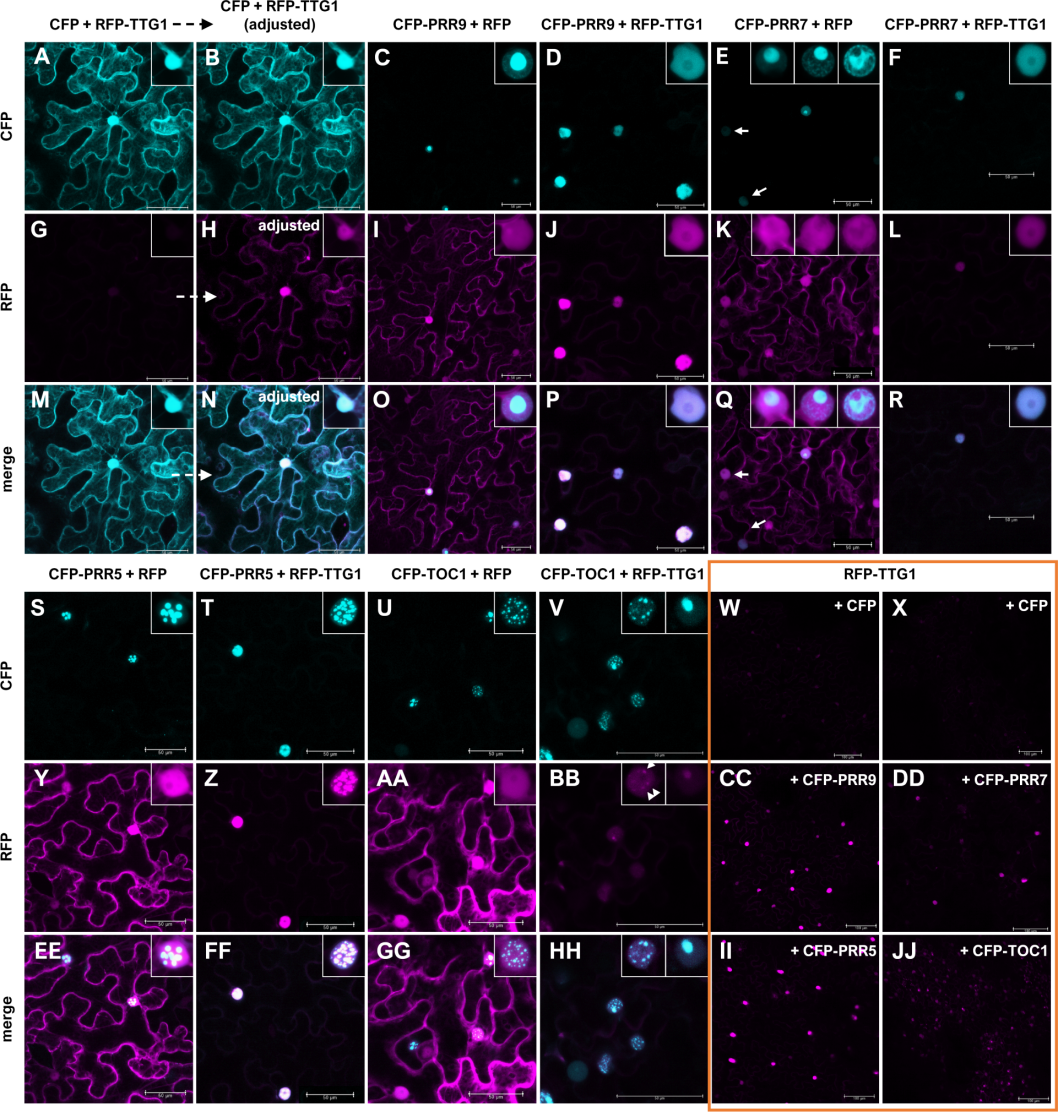
PRRs can re-localize TTG1 to different distinct (sub-)nuclear localizations. Representative, sequentially scanned and merged confocal stacks of *N. benthamiana* leaf epidermal cells. Combinations of RFP-TTG1 or RFP with CFP-PRR and CFP given above each column were co-expressed: CFP/RFP-TTG1 (A,B,G,H,M,N,W,X), CFP-PRR9/RFP (C,I,O), CFP-PRR9/RFP-TTG1 (D,J,P,CC), CFP-PRR7/RFP (E,K,Q), CFP-PRR7/RFP-TTG1 (F,L,R,DD), CFP-PRR5/RFP (S,Y,EE) CFP-PRR5/RFP-TTG1 (T,Z,FF,II), CFP-TOC1/RFP (U,AA,GG) and CFP-TOC1/RFP-TTG1 (V,BB,HH,JJ). The CFP- (A-F, S-V) and RFP-channel (G-L, W-DD, II, JJ) were merged (M-R, EE-HH) and a brightness-contrast correction was only applied for the CFP/RFP-TTG1 combination in B, H and N to visualize RFP-TTG1 presence and localization. Insets show representative observed nuclear localization. Please note that for CFP-PRR7/RFP (and to a minor extend for CFP-TOC1/RFP-TTG1) different subnuclear localizations were seen in all three experiments conducted and that the subnuclear localization of PRR9 and PRR7 was also modified by RFP-TTG1. White arrows for CFP-PRR7/RFP (E,Q) point to week CFP fluorescing nuclei without intense subnuclear foci. These were not dominant but present. For the CFP-TOC1/RFP-TTG1 combination (BB), arrowheads in the left inset of a characteristic nucleus point to faint subnuclear foci into which RFP-TTG1 is recruited. Pictures of all nuclei, including bars for those which are shown as insets, are shown in Fig. S8. Orange box: Non-adjusted pictures of the RFP channel detection for co-infiltrations of RFP-TTG1 with CFP or CFP-tagged PRRs showing an area of the respective leave. See also Fig. S9 for pictures with increased brightness and contrast. Bars equal 50 µm for representative cells and 100 µm for the leaf areas.

The TTG1 protein is cell-to-cell mobile (Bouyer et al., 2008). Knowing that nuclear trapping by GL3 is a relevant mechanism for TTG1 function in trichome patterning (Balkunde, Bouyer & Hulskamp, 2011; Bouyer et al., 2008), we analyzed the localization of TTG1 in presence and absence of PRR5 and bHLH92 and vice versa (Figs. 5C–5U, Figs. S8–S9). As reported before, tagged TTG1 is localized in the cytoplasm and in nucleus in epidermal cells of infiltrated tobacco leaves (e.g., Bouyer et al., 2008). PRR5 is only localized to the nucleus where it forms nuclear foci. When co-expressing RFP-tagged TTG1 and YFP-tagged PRR5, RFP-TTG1 localized predominantly in the nucleus where it co-localized with YFP-PRR5 (Figs. 5C–5K, Figs. S8–S9). In case of bHLH92, we obtained a different result. YFP-bHLH92 is enriched in the nucleus but also localizes to the cytoplasm when co-expressed with RFP alone. When co-expressed with RFP-TTG1, TTG1 localization did not change but the nuclear enrichment of bHLH92 did not occur (Figs. 5L–5U, Figs. S8–S9). We repeated the experiment with each of the PRRs (PRR5, PRR7, PRR9 and TOC1/PRR1) being fused to CFP in combination with RFP alone or RFP-TTG1 (Fig. 6, Figs. S8–S9). The PRRs did not only recruit TTG1 to the nucleus, TTG1 also changed the subnuclear localization for PRR9 and PRR7.

## Discussion

TTG1 is a pleiotropic regulator of early developmental traits in *A. thaliana*. Here we show, that this view has to be extended as TTG1 also acts later in the plant’s life cycle as a regulator of flowering time in *A. thaliana*.

The four used *ttg1* mutants in Col-0 background flowered earlier and overexpressors in the same background flowered later at long-day conditions than the respective wild type. This is consistent with an observed suppression of *FT* and *SOC1* transcript levels upon TTG1 overexpression. In our study, we followed the hypothesis that TTG1 acts upstream of *FT*. In this line, if a suppressor of *FT* is regulated by TTG1, its transcript level should be suppressed in *ttg1* mutants and increased in the TTG1 overexpressor line. For the mutants and overexpressors, we find a mixed bag of transcript profiles suggesting TTG1 to act in multilayered regulatory mechanisms.

### TTG1 variants, protein levels and the background

The L*er* wild type exhibited the earliest flowering wild type in our experiment which could impair an early flowering of *ttg1-1* relative to its wildtype with respect to time and number of leaves produced. Due to these results, it is not surprising that the flowering phenotype was not reported earlier as the *ttg1-1* has been heavily used in previous studies since the 1980s. The late flowering phenotype of *ttg1-10* in Ws background might be initially surprising but can be explained by the localization of the mutation within the *TTG1* gene. Floral buds did not express the *TTG1* transcript in this mutant (Larkin et al., 1999). This might deviate at different developmental stages and tissues as *ttg1-10* is an EMS mutant with a point mutation in the *TTG1* promoter. The mutation might change the expression pattern of TTG1, which in turn can suppress flowering. Also, a manifested second site mutation cannot be excluded as well as an effect of the Ws background.

Interestingly, the *ttg1-21* mutant, the presumably strongest mutant analyzed in this study, showed the mildest effect on flowering time as compared to the wildtype. At the warm condition, a significant deviation from the wild type could not be concluded for this mutant in terms of time. This suggests that the properties of the TTG1 protein might have a stronger effect on flowering time regulation than the reduced level or absence of TTG1. Presence of the TTG1 protein is not required for the plant to flower but elevated levels and variants can modulate flowering time in *A. thaliana*. Therefore, an overexpression line seems to be more beneficial to initially identify targets and interaction partners for TTG1-dependent regulation within the flowering time regulatory pathway. Thereby, young plant material can be used to provide the basis for a broad embedding of TTG1 in this pathway. The phenotype of the mutants is in agreement with the phenotype of the overexpression lines in the same background. However, TTG1-dependent regulation through protein level and type of protein variant might follow overlapping as well as differing regulatory mechanisms which might be dependent on the respective background and variant-specific interaction patterns to be dissected in the future.

### The developmental stage and age pathway

Our results acquired along the chronological and developmental axis point to an involvement of TTG1 in modulating the plastochron. Detailed meristem analysis at and around the time point of flowering are required in the future. The developmental stage and a possible tissue-specificity might also explain the *FT* and *SOC1* transcript levels which were not significantly increased in *ttg1* mutants. As TTG1 is a factor required for cell fate determination (Galway et al., 1994), developmental stage, tissue and cell specific effects might occur. Therefore, older plants than the used seedlings and the analysis of tissue specific expression might be required in a more detailed future analysis. This would be of particular relevance for the age pathway and GA signaling which were not covered in this study.

Overlaps with the age pathway might occur at the level of the *SQUAMOSA BINDING PROMOTER BINDING PROTEIN-LIKE* (*SPL*)s which are suppressed by microRNA156. SPLs are involved in regulating trichome density at later stages e.g., at the stem (Yu et al., 2010), SPL9 activates *TRIPTYCHON* and *TRICHOMELESS1* and is thought to modulate trichome density thereby (Yu et al., 2010). Both R3-MYBs belong to the so-called inhibitors which compete in the MBW complex scenarios with R2R3-MYBs for bHLH factor binding (Balkunde, Pesch & Hulskamp, 2010; Esch et al., 2003; Wang et al., 2008; Wester et al., 2009). TTG1 itself was reported to interact with SPL4 and SPL5 in yeast (Ioannidi et al., 2016) and the mutant of another inhibitor, *enhancer of try and cpc* 3 (*etc3)*, exhibits a differential *FT* and *SOC1* regulation in 21d-old LD-grown plants as compared to the wild type which equals the time of its early flowering time phenotype (Wada & Tominaga-Wada, 2015).

### WERWOLF (WER) and FT mRNA stability

If TTG1 acts at the *FT* gene, TTG1 can either act on the promoter or on other regulatory regions of *FT* (and *SOC1*) or affect the *FT* mRNA stability as reported for WER, a TTG1 network component (Seo et al., 2011). Seo and co-workers found that the mutant of *WER* flowers late and the R2R3-MYB factor WER is required for *FT* mRNA stability. The role of *WER* towards flowering time regulation was found to be independent of *CO* and *FLC*. Together with our results, the role of TTG1 and WER would be opposing which is not in line with a joined regulation following the classical MBW complexes regulatory mechanisms. However, we can not exclude that *FT* mRNA stability is changed in dependence of TTG1 complexes. By forming an MBW complex with WER, TTG1 could prevent WER from its function towards *FT* mRNA stability which would add to the late flowering time phenotype observed in TTG1 overexpressors.

### AP2-domain containing factors and the GA signaling branch

Several AP2-domain containing factors exhibit the expected tendencies to reduced transcript levels in *ttg1* mutants. However, the overexpressor lines did not show the respective opposing effect. Hence, an intact TTG1 seems to be required for normal circadian transcript profiles of these AP2-domain containing factors.

At the transcriptional level, TEM1 and TEM2 act as repressors of the TTG1-MBW complex components GL1, an R2R3-MYB factor, and the bHLH factors GL3 and EGL3 while TTG1 transcript levels are not affected (Matias-Hernandez et al., 2016). On the one hand, TEM1 and TEM2 act in a cell type-dependent manner (Matiaz-Hernandez, 2016). Therefore, an effect of deviating cell fate and differentiation in the *ttg1* mutants can have additional effects. On the other hand, TEMs control GA accumulation and distribution in the leaf mesophyll. They also integrate the photoperiod and GA signaling pathway in LD and SD conditions (Castillejo & Pelaz, 2008; Matiaz-Hernandez, 2016; Osnato et al., 2012). At the molecular level, reduced *TEM2* transcript levels might circumvent *FT* and *SOC1* transcript levels and cause an early flowering of *ttg1-9*. Reduced TEM2 levels lead to elevated GA levels which promote flowering time. The bHLH factors GL3, EGL3 and the R2R3-MYB factor GL1, can interact with the DELLA proteins RGA (REPRESSOR OF GA1-3 (mutant of *GA REQUIRING 1*)) 1 and RGA2 which both repress the transcriptional activation properties of the MBW complex. This suppression is derepressed by GA through GA-induced degradation of the DELLA proteins (Qi et al., 2014). Therefore, different binding properties of the mutant protein variants might cause additional regulatory loops through the GA signaling pathway to play a role.

### Competitive scenarios modulating CO protein levels

AP2-domain containing factors can bind directly to the *FT* promoter (Castillejo & Pelaz, 2008; Mathieu et al., 2009; Zhang et al., 2015). In addition, at the protein level, TOEs can interact with CO and thereby prevent CO from activating *FT* transcription (Zhang et al., 2015). In contrast, PRRs can stabilize the CO protein (Hayama et al., 2017). Although a reduction of *CO* transcript levels upon TTG1 overexpression at ZT12 is observed with similarities to the patterns of PRR overexpressors (Hayama et al., 2017), a leading role of CO in the TTG1-dependent regulation of *FT* transcript levels cannot be concluded based on the CO transcript levels. Nevertheless, at the protein level, TTG1 might either decrease CO protein levels or inactivate the CO protein and, thereby, reduce a CO-mediated *FT* activation. The interaction of TTG1 with PRRs and re-localization of PRRs, as suggested by our results, could have such an effect.

CO protein levels are elevated in *cop1-4* background (Jang et al., 2008). Overexpression of *TTG1* can also delay flowering time in *cop1-4* background. This indicates that either the TTG1 protein levels were sufficient to counteract the CO protein function at the protein level or that the TTG1 function can be or is mainly independent of CO. However, the effects on flowering time are difficult to compare among wild type and *cop1-4* background as we found increased *TTG1* transcript levels in *cop1-4*.

### LWDs, TTG1 and the clock

Further upstream in the photoperiodic pathway, LWD proteins act as activators within the loops of the circadian clock (Shim, Kubota & Imaizumi, 2017). It is conceivable that a partial overlap in function exists as these are the two closest homologs of TTG1 in *A. thaliana* but also differences are expected. Towards this end, a detailed evolutionarily focused analysis will be of interest.

While *LWD1* transcript levels show a strong circadian response with highest levels late at night and in the long-day morning, *LWD2* and, in our results, *TTG1* do not show this pattern and remain at a similar level during the day and night (Wu, Wang & Wu, 2008). Interestingly, promoter-luciferase constructs showed rhythmic activity of both *LWD* promoters (Wang et al., 2011). With a focus on LWD1, its binding in a time dependent manner at the promoters of *PRR5, PRR7*, *PRR9* and *CCA1* was revealed (Wang et al., 2011). In *lwd1lwd2* double mutants, *CCA1* and *LHY* transcript levels are reduced, the period is shortened and shifted forward (Wu, Wang & Wu, 2008). In the late afternoon to early morning at LD condition, TTG1 overexpression increases the transcript levels of *PRR5*, *PRR7*, *PRR9*, *CCA1* and *LHY* and potentially reduces the respective transcriptional amplitudes.

CCA1 expression can be activated by LWD1 which acts together with interacting TCP transcription factors (Wang et al., 2011; Wu et al., 2016). TTG1 might act similarly but, also, a mechanism as suggested for CO is possible for CCA1 and LHY. Through binding of TTG1 to PRRs, these might no longer be able to form a complex with the transcriptional repressors TOPLESS/TOPLESS RELATED PROTEINs at the *CCA1* and *LHY* promoters (Wang, Kim & Somers, 2013). Consequently, *CCA1* and *LHY* transcript levels would increase and the circadian period would be lengthened (Wang, Kim & Somers, 2013). We observed respective transcript level characteristics upon TTG1 overexpression apart of the period which was not tested.

Differential complex composition of TTG1 with interacting transcriptional activators and repressors might act in comparable regulatory mechanisms mediating target specific DNA-interaction. PRRs and bHLH92 described in this study are excellent candidates. As we did not find strong indications for a clear interaction with LWDs, mechanisms related to PRRs and bHLH92 at the protein level are likely to differ between the TTG1 and the LWDs. Being a bHLH factor, bHLH92 might fit well into the regulatory scheme known for TTG1. This would require the identification of an R2R3-MYB interacting with bHLH92.

### TTG1 might act through TTG1-PRR and TTG1-FLC-FT modules (Fig. 7, Fig. S10)

The subnuclear localization patterns upon co-expression of PRRs and bHLH92 with TTG1 can provide a regulatory mechanism. Does this have an influence on transcriptional activation? What is the identity of the subnuclear foci? Are the interaction partners binding in concert to specific loci, stabilize each other, are they stored or deactivated within these foci or are competitive scenarios at work? These are pressing questions to be answered.

Through such mechanisms, known trait networks of the clock, like those of the PRRs, can be linked with the TTG1 trait network. For PRR5, target promoters were identified which comprise different transcription factors involved e.g., in auxin production, hypocotyl growth and cold-stress response. This might be intermingled with traits and temperature responsiveness in TTG1-dependence. TTG1 might suppress PRR target modulation on relevant downstream targets of both factors at a respective developmental stage. *FT* might be such a target as it was shown that a PRR-CDF-FT module can exists. PRR5 can directly suppress CDF expression (Nakamichi et al., 2012) and CDFs can suppress *FT* at its promoter (Song et al., 2012). A TTG1-PRR-CDF-FT module could bypass *GI* and *CO* and could link TTG1 effects on clock gene modulation with *FT* suppression in a competitive scenario with PRR-CDF-FT modules.

**Figure 7:**
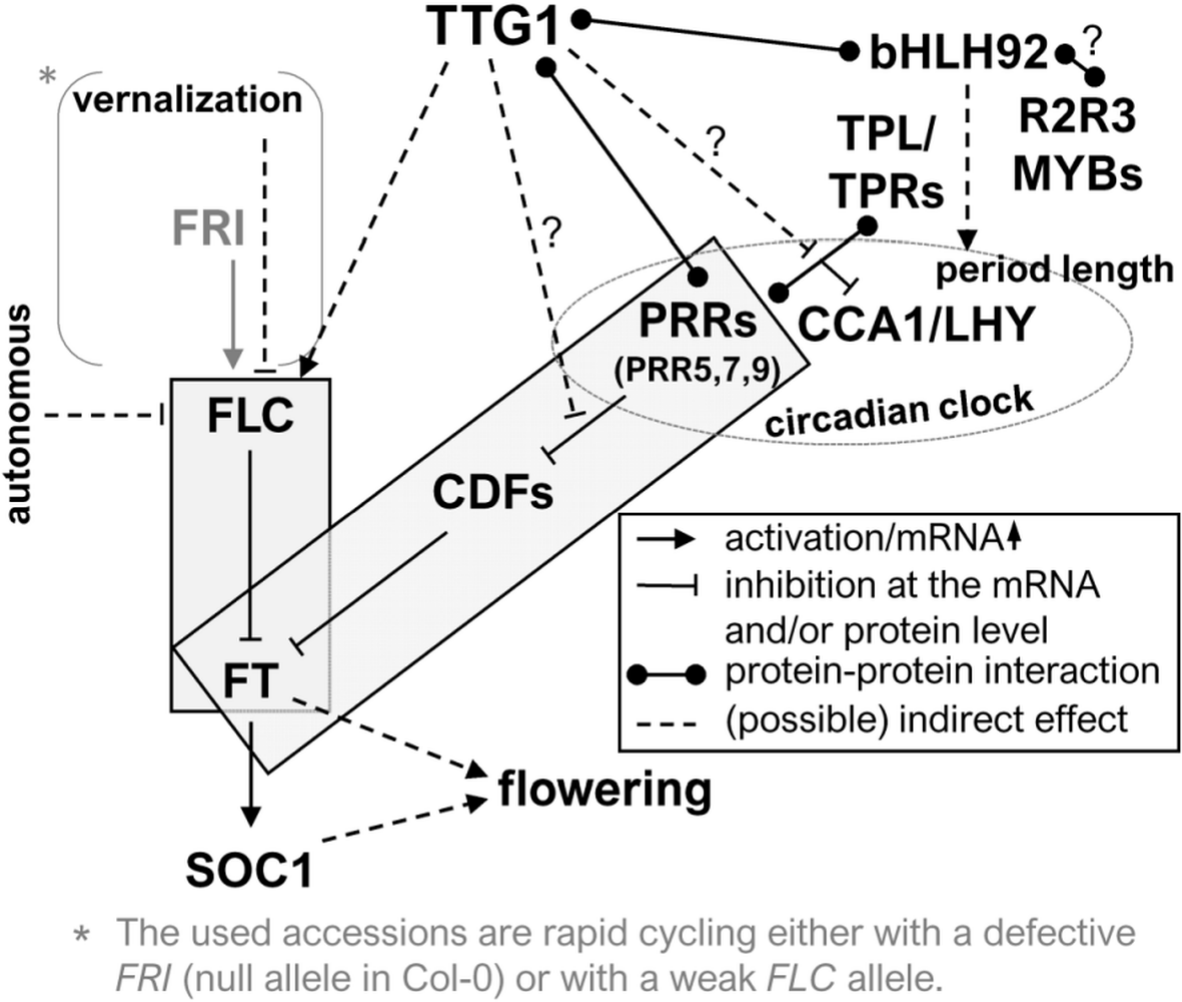
Model. The model embeds TTG1 in the known flowering time regulation. Boxes: discussed modules explaining obtained flowering time results. See the discussion for more details. A more detailed description of the pathway is also provided in the introduction and can be found e.g., in Blumel, Dally & Jung (2015).

Our results suggest a possible, parallel TTG1-FLC-FT module. Elevated *FLC* transcript levels lead to an increase in FLC-mediated *FT* and *SOC1* suppression and consequently to late flowering in the overexpression line. In line with this role of TTG1, the weak allele of *FLC* in L*er* background could explain the absence of a flowering time phenotype in the strong and heavily used *ttg1-1* mutant. Therefore, when anlalyzing this mutant, a role of TTG1 towards flowering time regulation was not detectable before. All used accessions in this study are rapid cycling accessions lacking a functional FRI allele and therefore immediately exposing modulations at *FLC* to potential phenotypic detection.

### Overlapping regulatory network

The annual plant *A. thaliana* completes its life cycle with the production and ripening of seeds and enters the new life cycle following seed dormancy with seed germination. The reproductive success depends therefore on the appropriate timing of flowering and seed ripening as well as germination thereafter. Therefore, it is not surprising that a pleiotropic regulator like TTG1 which is strongly involved in the regulation of various relevant seed traits, is also involved in the regulation of flowering time. Here, it is noteworthy that TTG1-dependent gene regulatory network components including TTG1 have the potential to intervene in several sub-pathways of flowering time regulation. We found that TTG1 can even overwrite the transcriptional scenario in *cop1-4* in regard to the floral integrators FT and SOC1. Moreover, TTG1 variants are likely to be of relevance in adaptation to temperature seasonality, minimum temperature and daylength (Hancock et al., 2011). This strongly suggests an adaptive value of the TTG1-dependent trait network which is strengthened by the overlapping gene regulatory networks of TTG1 and flowering time regulation as substantiated with this study.

## Conclusions

Plants can respond to endogenous and exogenous cues through concerted regulation of specific trait networks. Pleiotropic regulators can aid to reveal such trait networks of adaptive value. The pleiotropic regulator *TRANSPARENT TESTA GLABRA 1* is known as head of a conserved gene regulatory network regulating early developmental traits. Surprisingly little has been known about its involvement in late developmental trait regulation. We reveal that TTG1 is a flowering time regulator in *Arabidopsis thaliana* and initially embedded TTG1 in the flowering time regulatory pathway. TTG1 modulates transcript levels of key elements within this pathway—the floral integrators *FT* and *SOC1*. We show that TTG1 might act upstream of *FLC* and the circadian clock. At the protein level we found differential interdependencies with regard to the subcellular and subnuclear localization of clock proteins and TTG1 *in planta*. In summary, our results provide an initial embedding of TTG1 in the flowering time regulatory pathway.

##  Supplemental Information

10.7717/peerj.8303/supp-1 Figure S1 Characterization of *ttg1-21* and *ttg1-22*

10.7717/peerj.8303/supp-2 Figure S2 Alignment of *TTG1*, *LWD1* and *LWD2* CDS containing the primer binding sited for “TTG1 both” and “TTG1 no LWD” qRT-PCR experiments

10.7717/peerj.8303/supp-3 Figure S3 Additional blots and analysis of protein levels in TTG1 overexpressor lines

10.7717/peerj.8303/supp-4 Figure S4 Circadian profiles of endogenous and overexpressed *TTG1* transcript levels

10.7717/peerj.8303/supp-5 Figure S5 Similar to *CO*, *GI* transcript levels cannot explain the significant reduction of *FT* transcript levels in TTG1 overexpression lines

10.7717/peerj.8303/supp-6 Figure S6 Flowering time analysis of *TTG1* gene regulatory network bHLH factor mutants and respective overexpression lines in *A. thaliana* Col-0 background at warm long day conditions

10.7717/peerj.8303/supp-7 Figure S7 Y2H interaction test

10.7717/peerj.8303/supp-8 Figure S8 PRRs can re-localize TTG1 to different distinct (sub-)nuclear localizations and TTG1 can prevent the nuclear enrichment of CFP-bHLH92 as revealed by CLSM of nuclei in epidermal cells of infiltrated tobacco leaves

10.7717/peerj.8303/supp-9 Figure S9 Epidermal areas of infiltrated tobacco leaves revealing the nuclear enrichment of RFP-TTG1 upon co-expression with PRRs and the abolished enrichment of CFP-bHLH92 by co-expression of RFP-TTG1

10.7717/peerj.8303/supp-10 Figure S10 Significant results (*P*< 0.5) from qRT-PCR experiments in the context of the relevant flowering time pathway components

10.7717/peerj.8303/supp-11 Table S1 Datalogger results from growth condition measurements

10.7717/peerj.8303/supp-12 Table S2 Results of phenotyping experiments and statistics

10.7717/peerj.8303/supp-13 Table S3 Results of qRT-PCR experiments for circadian TTG1, CO, FT, SOC1 transcript levels in overexpression lines and statistics

10.7717/peerj.8303/supp-14 Table S4 Qualitative analysis of YFP-fluorescence in the overexpression lines as seen and classified by eye at a fluorescence stereo microscope

10.7717/peerj.8303/supp-15 Table S5 Results of circadian qRT-PCR experiments and statistics

10.7717/peerj.8303/supp-16 Table S6 Results of Y2H screens

10.7717/peerj.8303/supp-17 Table S7 Used Mutants

10.7717/peerj.8303/supp-18 Table S8 Primers

10.7717/peerj.8303/supp-19 Table S9 TTG1, LWD1 and LWD2 CDS used for Fig. S2

## Additional Information and Declarations

### Data Availability

The following information was supplied regarding data availability: Raw data are available in the Supplemental Files.
