# Supplementary figures and images for: TRANSPARENT TESTA GLABRA 1 participates in flowering time regulation in Arabidopsis thaliana
Source: PeerJ. 2020 Jan 20;8:e8303. doi: 10.7717/peerj.8303 (PMC6977477; doi:10.7717/peerj.8303)

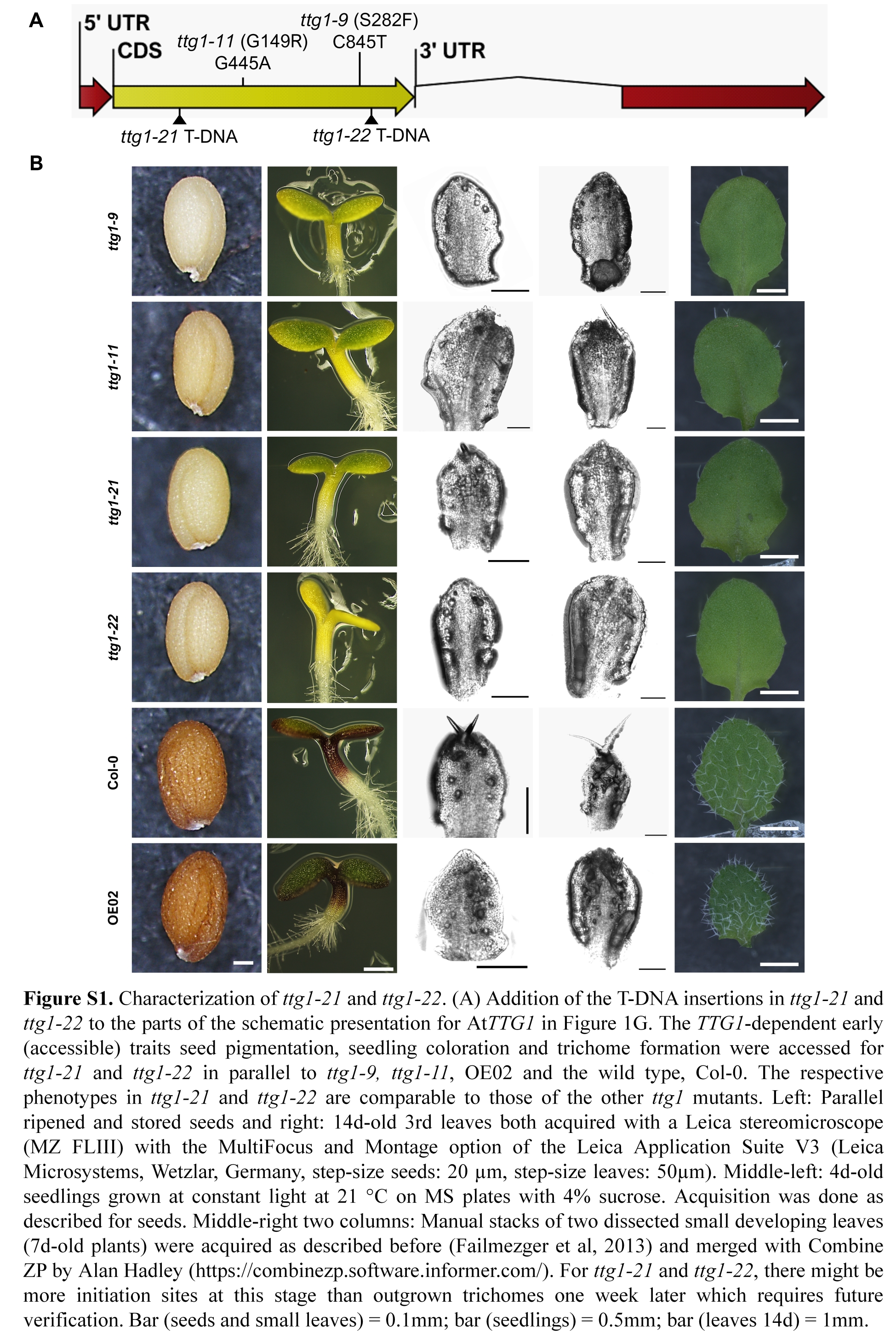

Supplement: Figure S1 [file peerj-08-8303-s001.jpg]

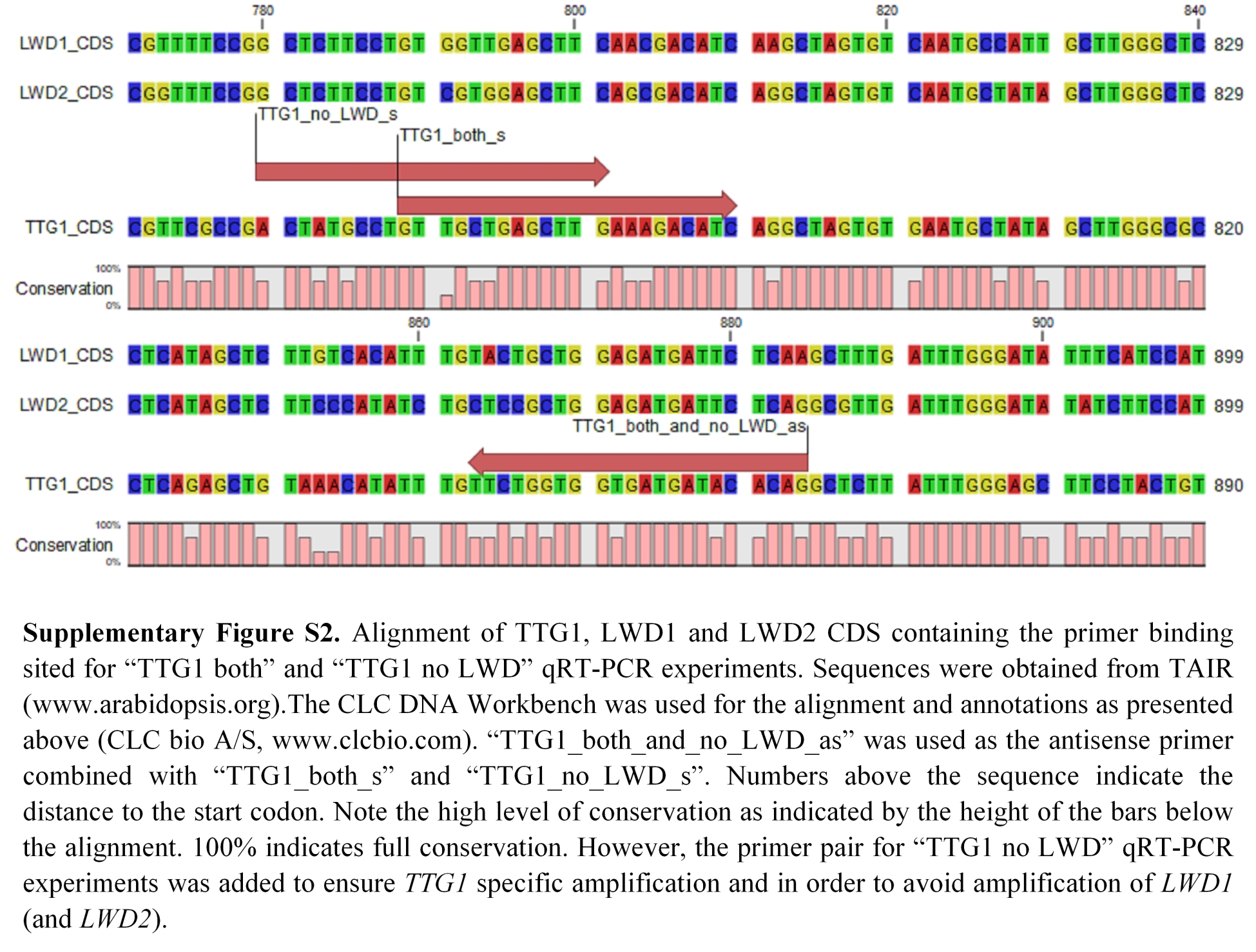

Supplement: Figure S2 [file peerj-08-8303-s002.jpg]

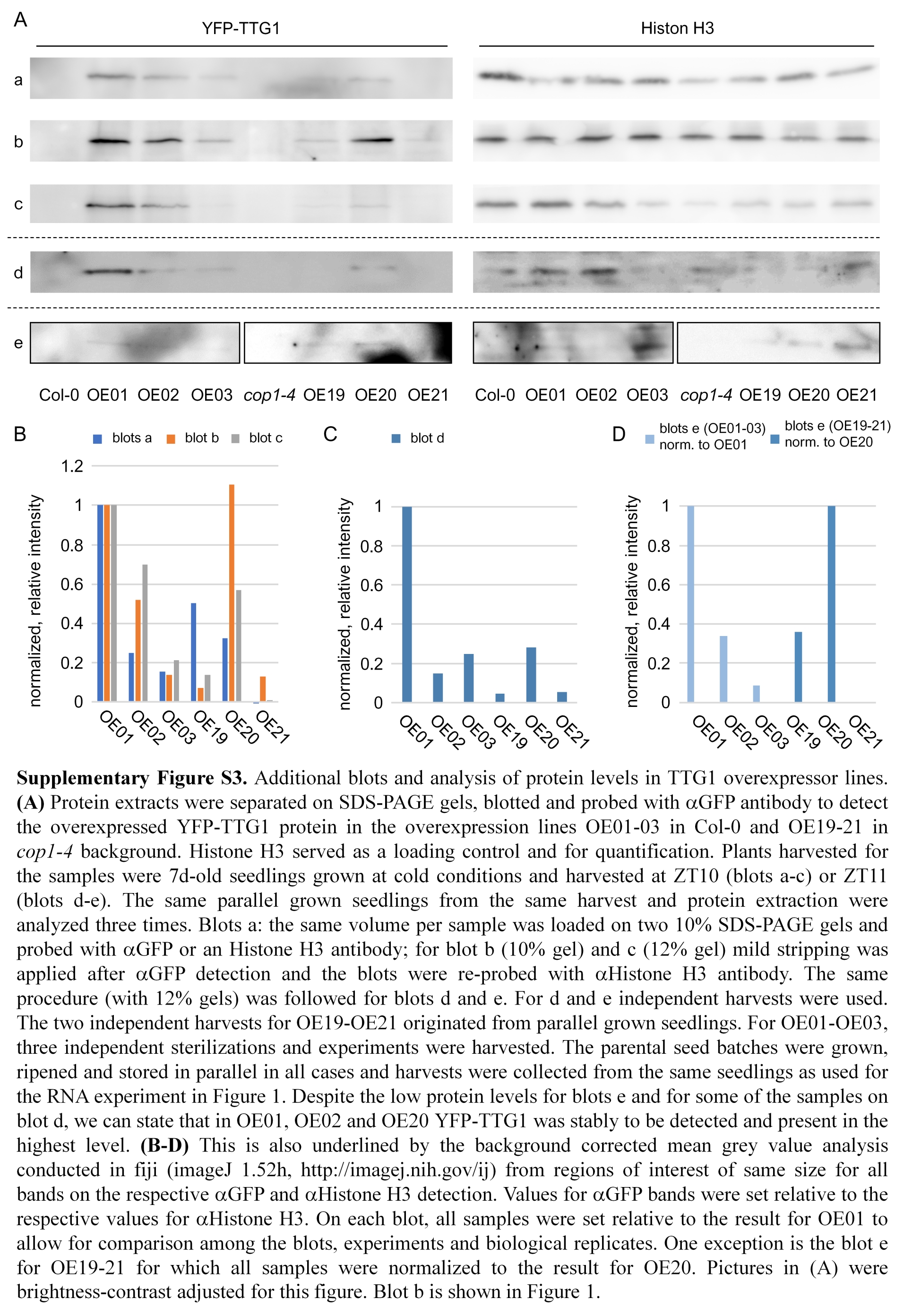

Supplement: Figure S3 [file peerj-08-8303-s003.jpg]

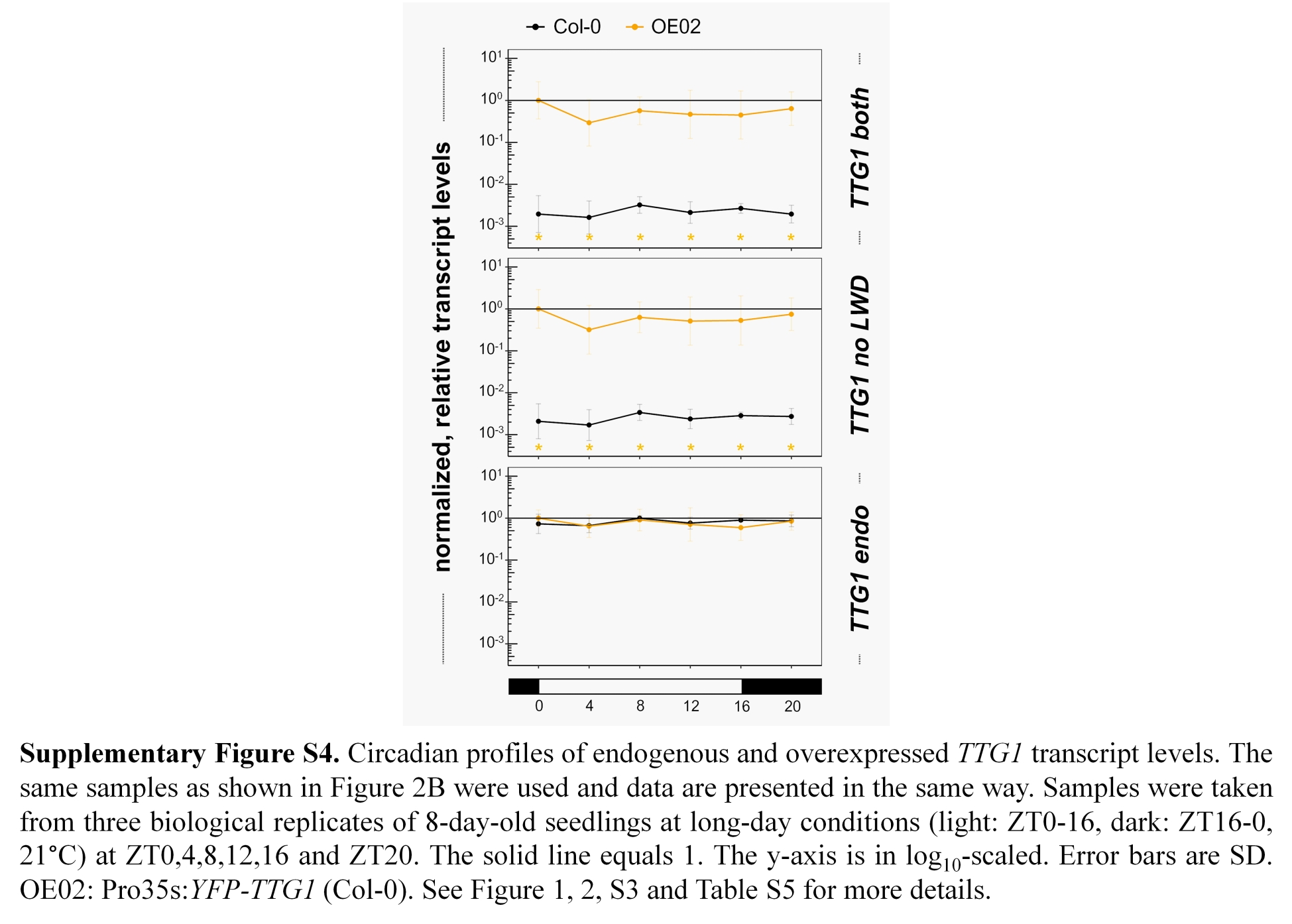

Supplement: Figure S4 [file peerj-08-8303-s004.jpg]

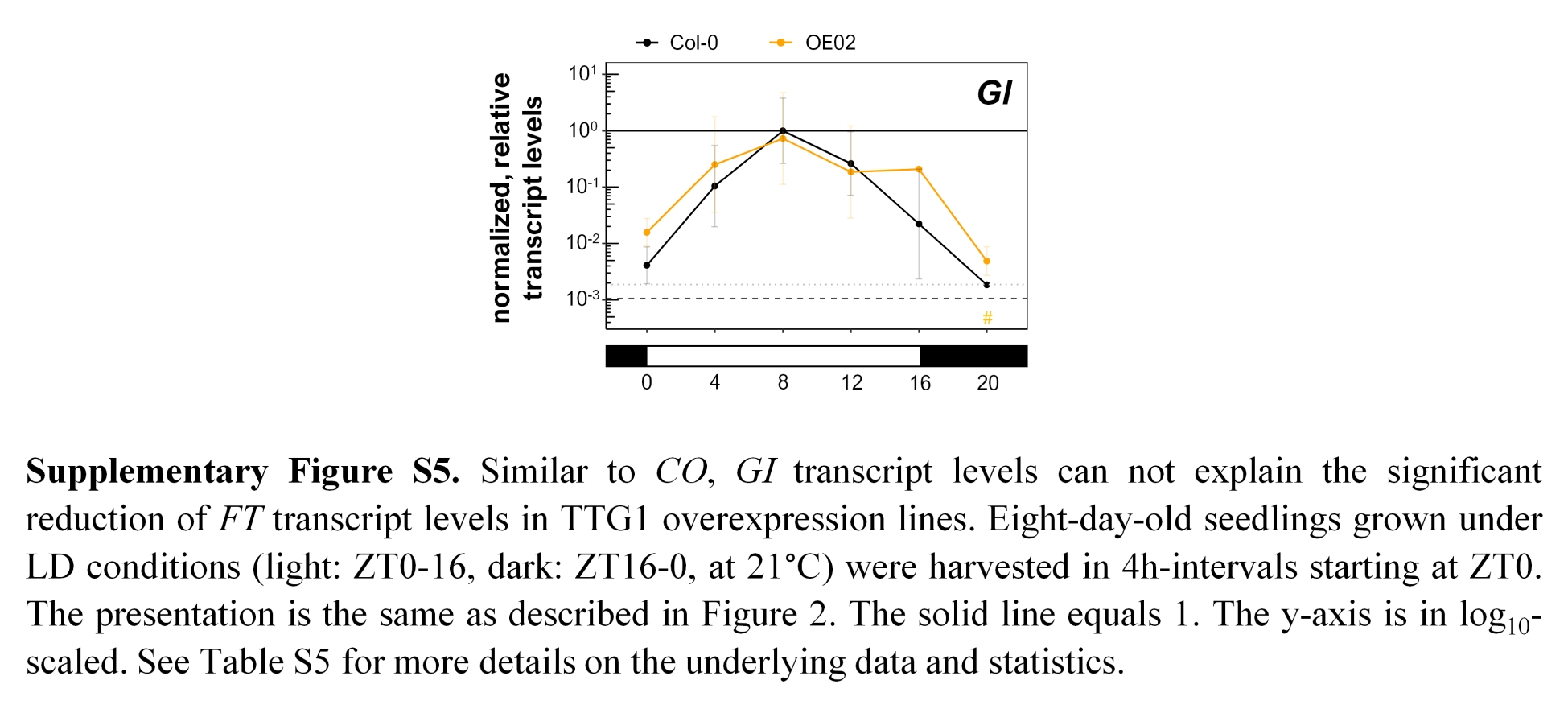

Supplement: Figure S5 [file peerj-08-8303-s005.jpg]

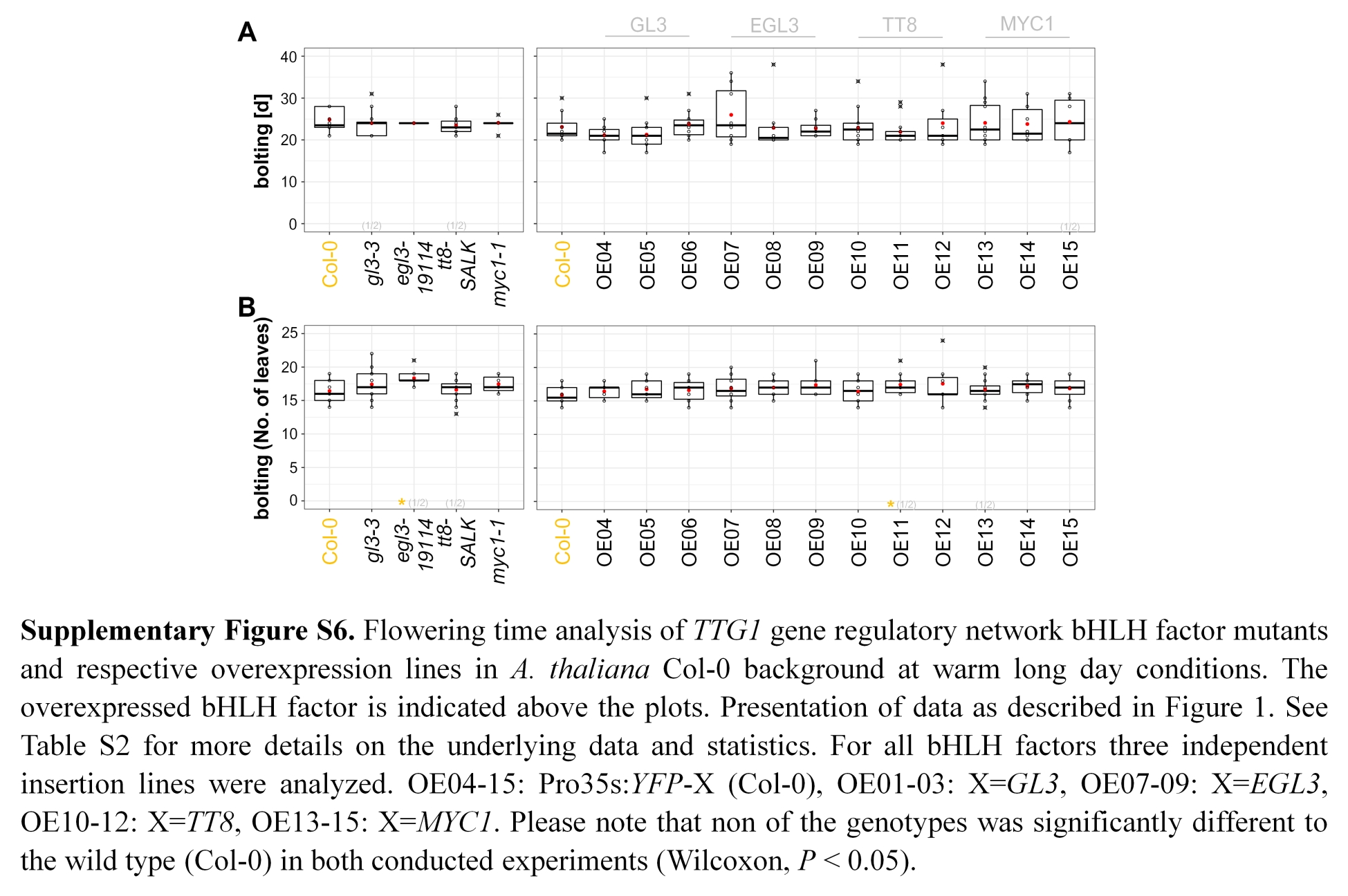

Supplement: Figure S6 [file peerj-08-8303-s006.jpg]

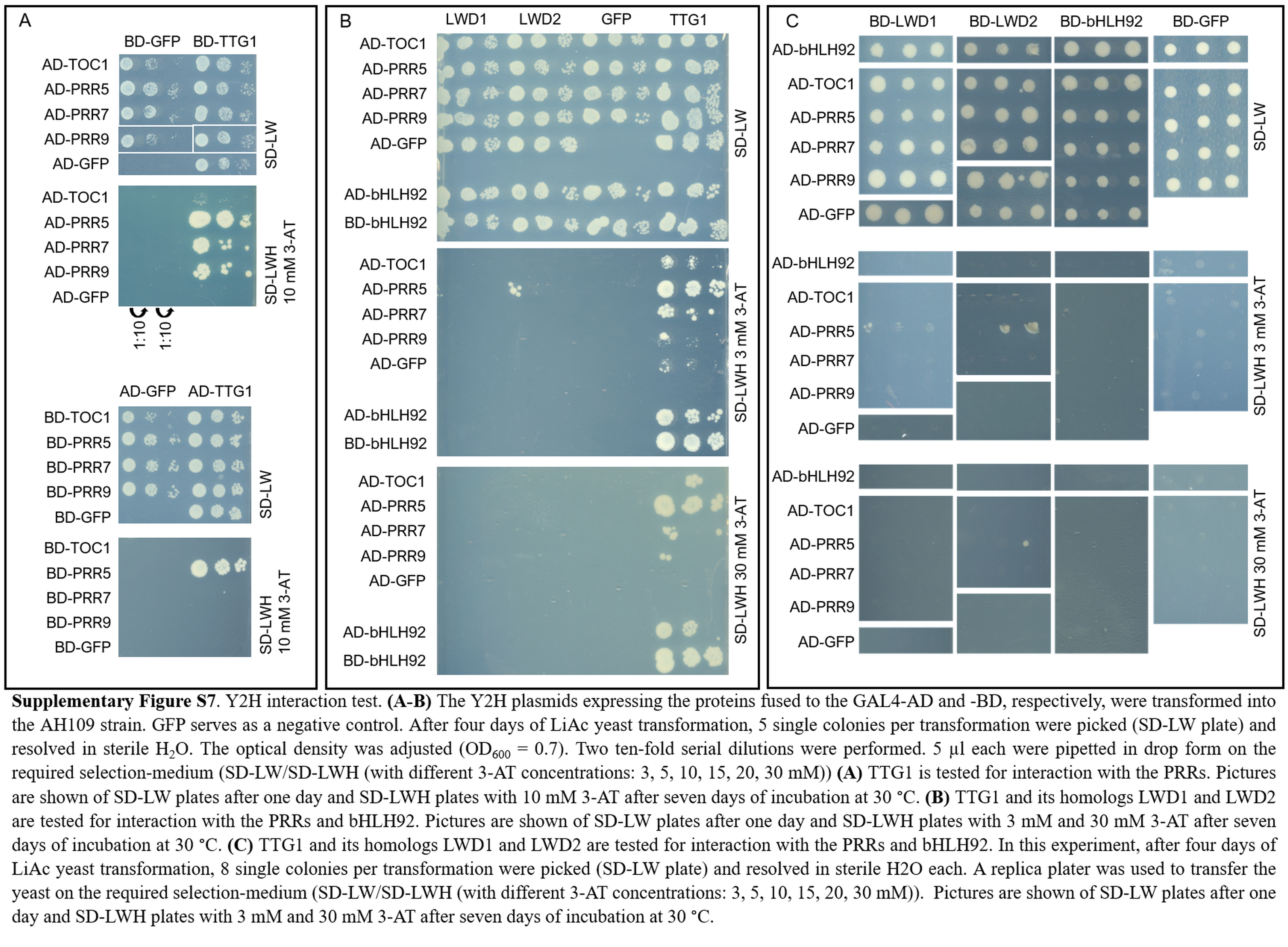

Supplement: Figure S7 [file peerj-08-8303-s007.jpg]

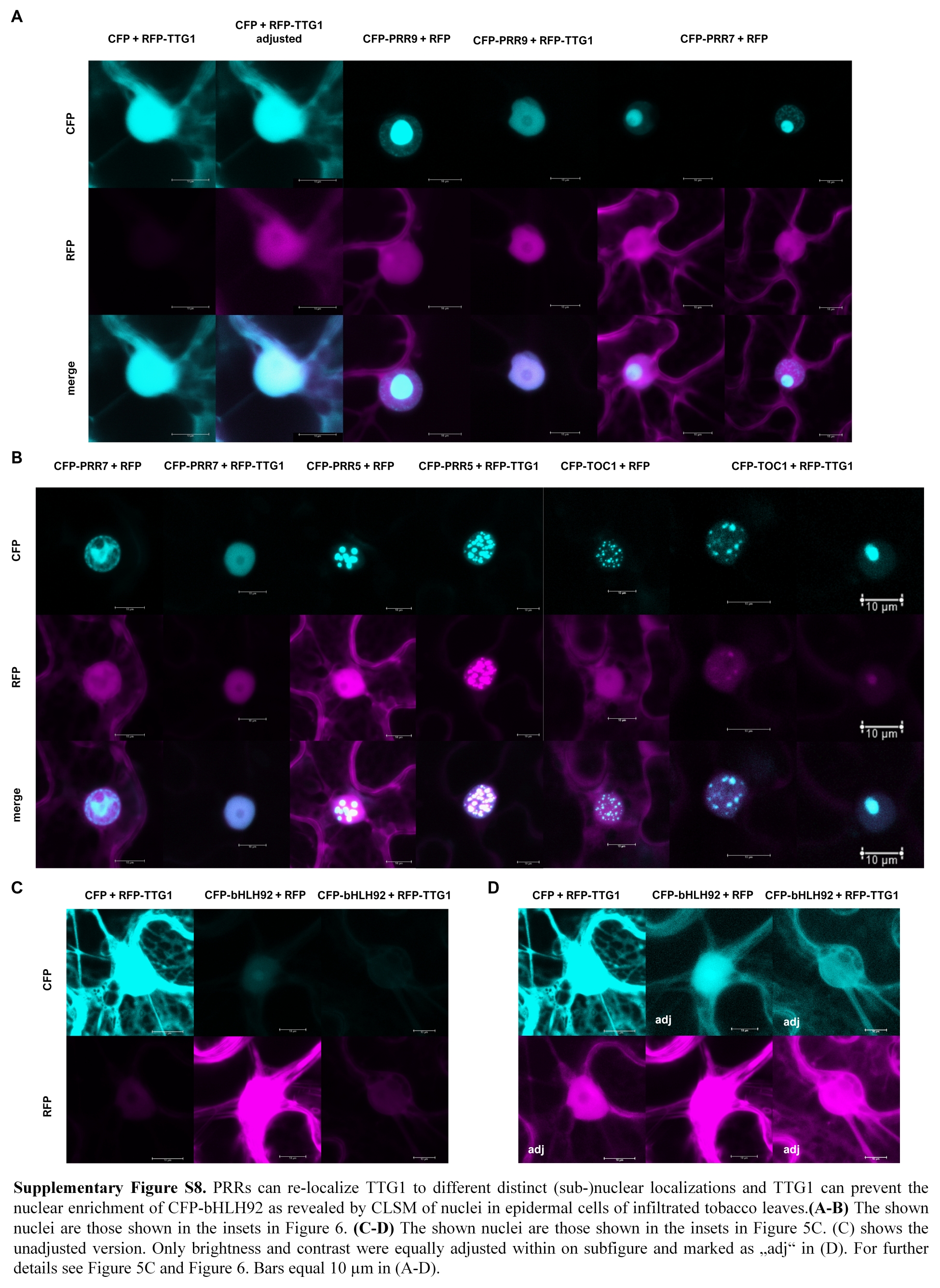

Supplement: Figure S8 [file peerj-08-8303-s008.jpg]

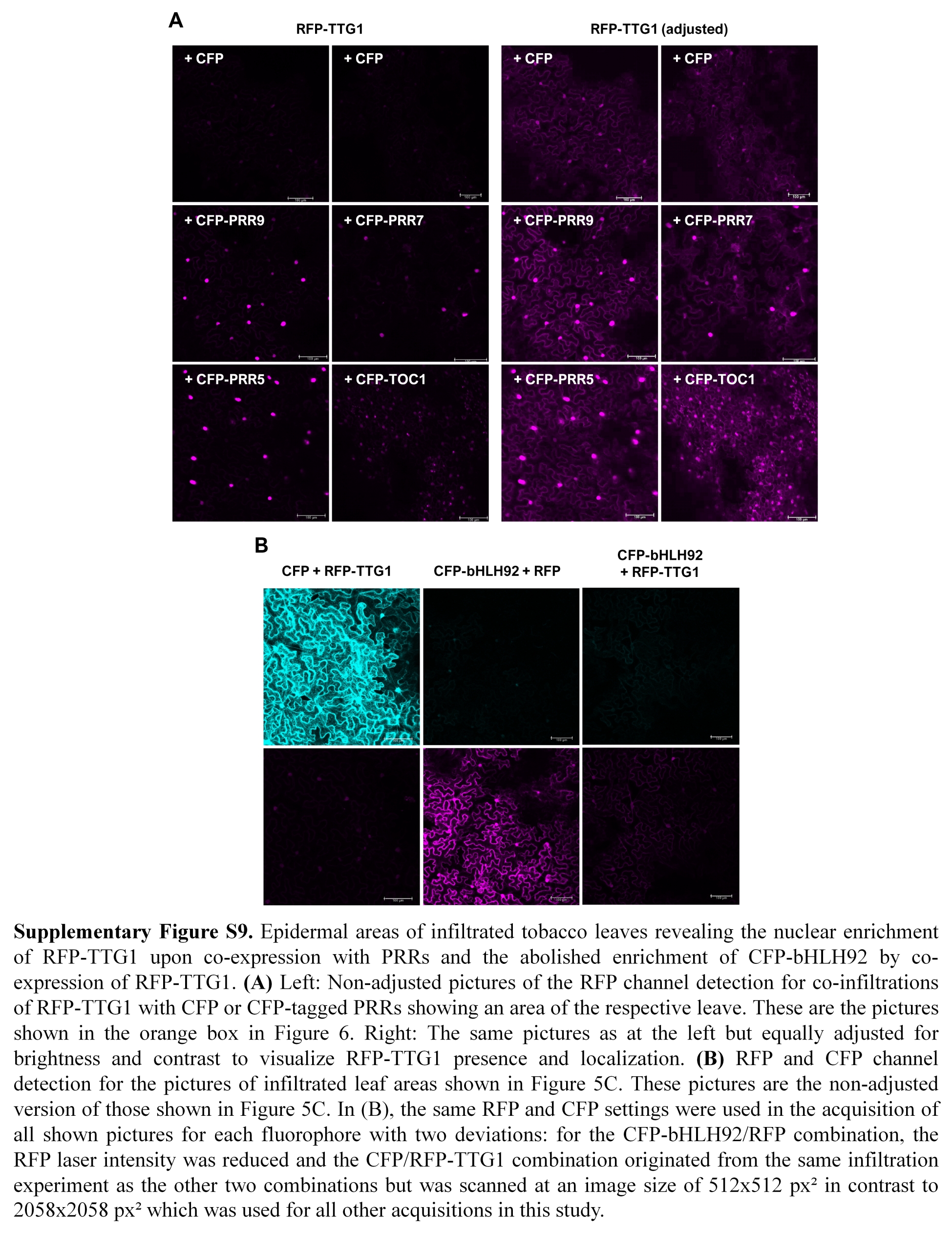

Supplement: Figure S9 [file peerj-08-8303-s009.jpg]

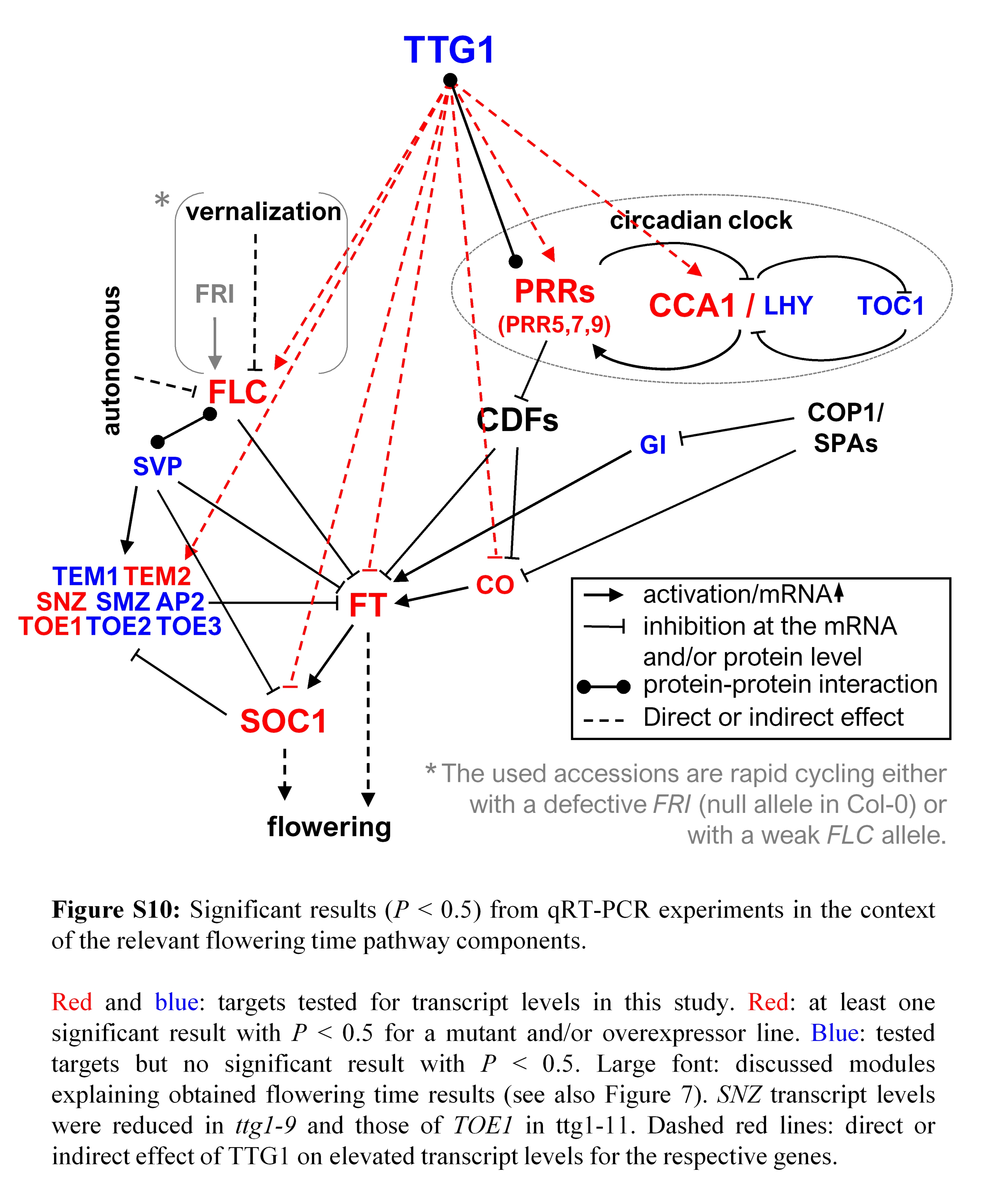

Supplement: Figure S10 [file peerj-08-8303-s010.jpg]
