# Supplementary material for: TRANSPARENT TESTA GLABRA 1 participates in flowering time regulation in Arabidopsis thaliana
Source: PeerJ. 2020 Jan 20;8:e8303. doi: 10.7717/peerj.8303 (PMC6977477; doi:10.7717/peerj.8303)
Supplement: Table S1 [file peerj-08-8303-s011.docx]

**Supplementary Table S1.** Datalogger results from growth condition measurements.

|  | **Temperature** | | |  | **Humidity** | | | **Light intensity** |
| --- | --- | --- | --- | --- | --- | --- | --- | --- |
| **condition** | **Day+night* [°C]** | **Day [°C]** | **Night [°C]** |  | **Day+Night* [%]** | **Day [%]** | **Night [%]** | **[µmol*m^-2^*s^-1^]** |
| LD “warm” - TTG1 experiments | 23.7 | 24.7 | 21.6 |  | 61.2 | 58.1 | 67.4 | 80-120 |
| LD “warm” ** - bHLH experiment 2 | 23.9 | 24.9 | 21.9 |  | n.d. | n.d. | n.d. | 80-120 |
| LD “cold” | 21.4 | 22.2 | 19.8 |  | 56.9 | 54.3 | 61.9 | 80-120 |
| LD “cold” - qRT-PCR area | 22.4 | 23.6 | 19.9 |  | 51.1 | 47.9 | 57.6 |  |

Available plant chambers at their given settings were used and the conditions were recorded. Light was set manually and regularly controled. Data logger: EBI 300 TH, calibrated; data were extracted with the software Winlog.basic 2.8 (both www.ebro.com, Xylem Analytics Germany Sales GmbH & Co. KG). The datalogger was positioned at the level of the rosettes or plates, respectively, and not covered by plants. * For phenotyping, at least one day-night cycle at the front (center of the walk-in plant chamber) and one at the back (wall) per used shelf was recorded and the weighted mean for the respective condition is given (LD: 16h light/8h dark, SD: 8h light/16h dark) is given. Subsequently, a thermometer was used to controle the temperature at the plant’s level. Every used shelf was measured for 5 (“cold”) or 7 (“warm”) consequitive days. ** same walk-in chamber, measurement repeated. Light was measured with the same device in both chambers. Light sources: OSRAM L 58W/840 LUMILUX Cool White.
