# Supplementary material for: TRANSPARENT TESTA GLABRA 1 participates in flowering time regulation in Arabidopsis thaliana
Source: PeerJ. 2020 Jan 20;8:e8303. doi: 10.7717/peerj.8303 (PMC6977477; doi:10.7717/peerj.8303)
Supplement: Table S4 [file peerj-08-8303-s014.docx]

**Supplementary Table S4.** Qualitative analysis of YFP-fluorescence in the overexpression lines as seen and classified by eye at a fluorescence stereo microscope.

|  |  | **No. of plants with the following fluorescence category** | | | | | |  |
| --- | --- | --- | --- | --- | --- | --- | --- | --- |
| **genotype** | **condition** | **0** | **0-1** | **1** | **2** | **2-3** | **3** | **sum** |
| OE01 | cold | 1 (1) | 0 | 0 | 15 (2) | 0 | 10 (1) | 26 (4) |
| **OE02** | cold | 0 | 0 | 1 | 6 | 7 | 12 | **26 (0)** |
| OE03 | cold | 1 | 0 | 0 | 19 (10) | 0 | 2(1) | 22 (12) |
| OE19 | cold | 22 | 6 | 0 | 0 | 0 | 0 | 28 (0) |
| **OE20** | cold | 0 | 0 | 0 | 2 | 0 | 19 | **21 (0)** |
| OE21 | cold | 0 | 1 | 2 | 10 (5) | 0 | 1 | 14 (5) |
| OE01 | warm | 0 | 0 | 0 | 4 (1) | 0 | 8 | 12 (1) |
| **OE02** | warm | 0 | 0 | 0 | 24 (1) | 0 | 0 | **24 (1)** |
| OE03 | warm | 0 | 0 |  | 11 (8) | 0 | 0 | 11 (8) |
| OE19 | warm | 6 | 0 | 0 | 0 | 0 | 0 | 6 (0) |
| **OE20** | warm | 0 | 0 | 0 | 6 | 0 | 0 | **6 (0)** |
| OE21 | warm | 1 | 2 (1) | 3 | 0 | 0 | 0 | 6 (1) |

Fluorescence category 0: no observable YFP-fluorescence by eye. 3: strongest YFP-fluorescence observed. Qualitative analysis of YFP fluorescence using a fluorescence stereo microscope was assessed for plants of different age: plants analyzed from the cold condition were either 44 or 55 days in the plant chamber, plants from the warm condition were either 43 or 55 days (OE01-03) or either 34 or 35 days (OE19-21) in the chamber. On every day of analysis plants from all overexpressors within one background were analyzed. In brackets: for this number of plants (e.g. for 15 (2): for 2 out of 15 plants) with this assigned intensity category, one of the following observations was made: the fluorescence appeared patchy either within individual leaves or the whole plant, the plant's center of the rosette was missing fluorescence, when no fluorescence intensity was assigned the leaves were in addition glabrous. All these scenarios suggest that silencing occurred in these plants. **Bold:** these lines were selected for the analysis of relative transcript levels.
