# Supplementary material for: TRANSPARENT TESTA GLABRA 1 participates in flowering time regulation in Arabidopsis thaliana
Source: PeerJ. 2020 Jan 20;8:e8303. doi: 10.7717/peerj.8303 (PMC6977477; doi:10.7717/peerj.8303)
Supplement: Table S6 [file peerj-08-8303-s016.docx]

**Supplementary Table S6.** Results of Y2H screens.

| **AGI** | **Symbol** | **Name or Description** |
| --- | --- | --- |
| AT1G13390 | NA | translocase subunit seca |
| AT1G55210 | NA | Disease resistance-responsive (dirigent-like protein) family protein |
| AT1G63650 | EGL3 | ENHANCER OF GLABRA 3 |
| AT1G80620 | NA | S15/NS1%2C RNA-binding protein |
| AT2G18210 | NA | hypothetical protein |
| AT2G34250 | NA | SecY protein transport family protein |
| AT2G42760 | NA | DUF1685 family protein |
| AT3G42800 | NA | AF-like protein |
| AT3G47640 | PYE | POPEYE |
| AT5G08630 | NA | DDT domain-containing protein |
| AT5G16110 | NA | hypothetical protein |
| AT5G24470 | PRR5 | pseudo-response regulator 5 |
| AT5G43650 | BHLH92 | basic helix-loop-helix (bHLH) DNA-binding superfamily protein |
| AT5G61330 | NA | rRNA processing protein-like protein |

AGI, symbol, name or description were extracted from TAIR (www.arabidopsis.org).
