# Supplementary material for: TRANSPARENT TESTA GLABRA 1 participates in flowering time regulation in Arabidopsis thaliana
Source: PeerJ. 2020 Jan 20;8:e8303. doi: 10.7717/peerj.8303 (PMC6977477; doi:10.7717/peerj.8303)
Supplement: Table S7 [file peerj-08-8303-s017.docx]

**Supplementary Table S7.** Used Mutants

| **related gene** | **mutants/genotypes** | **AGI** | **mutation/insertion** | **background** | **species** | **citations** |
| --- | --- | --- | --- | --- | --- | --- |
| *TTG1* | *ttg1-1* | AT5G24520 | EMS | L*er* | *A. thaliana* | (Koornneef, 1981; Walker et al., 1999) |
| *TTG1* | *ttg1-9* | AT5G24520 | EMS | Col-0 | *A. thaliana* | (Walker et al., 1999) (Larkin et al., 1994) |
| *TTG1* | *ttg1-10* | AT5G24520 | EMS | Ws-2 | *A. thaliana* | (Larkin et al., 1994; Larkin et al., 1999) |
| *TTG1* | *ttg1-11* | AT5G24520 | EMS | Col-0 | *A. thaliana* | (Larkin et al., 1994; Larkin et al., 1999) |
| *TTG1* | *ttg1-21* | AT5G24520 | T-DNA^1^ | Col-0 | *A. thaliana* | (Rosso et al., 2003; Appelhagen et al., 2014) |
| *TTG1* | *ttg1-22* | AT5G24520 | T-DNA^1^ | Col-0 | *A. thaliana* | (Rosso et al., 2003; Appelhagen et al., 2014) |
| *COP1* | *cop1-4* | AT2G32950 | EMS | Col-0 | *A. thaliana* | (McNellis et al., 1994) |
| *GL3* | *gl3-3* | AT5G41315 | T-DNA^1^ | Col-0 | *A. thaliana* | (Rosso et al., 2003; Jakoby et al., 2008) |
| *EGL3* | *egl3-19114* | AT1G63650 | T-DNA^2^ | Col-0 | *A. thaliana* | (Alonso et al., 2003; Wester et al., 2009) |
| *TT8* | *tt8-SALK* | AT4G09820 | T-DNA^2^ | Col-0 | *A. thaliana* | (Alonso et al., 2003) |
| *MYC1* | *myc1-1* | AT4G00480 | T-DNA^2^ | Col-0 | *A. thaliana* | (Alonso et al., 2003; Pesch et al., 2013) |

Identifiers: AGI (The Arabidopsis Genome Initiative) ID: (www.arabidopsis.org) ^1^ origin: GABI (“Genomanalyse im biologischen System Pflanze”) -Kat (“Kölner Arabidopsis T-DNA”) lines (Rosso et al., 2003); ^2^ origin: SALK line (Alonso et al., 2003). Col: Columbia, L*er*: Landsberg *erecta*, Ws: Wassilewskaja, EMS: Ethyl methanesulfonate (used for mutagenesis), T-DNA: transfer-DNA

Alonso, J.M., Stepanova, A.N., Leisse, T.J., Kim, C.J., Chen, H., Shinn, P., et al. (2003). Genome-wide insertional mutagenesis of Arabidopsis thaliana. *Science* 301(5633)**,** 653-657. doi: 10.1126/science.1086391.

Appelhagen, I., Thiedig, K., Nordholt, N., Schmidt, N., Huep, G., Sagasser, M., et al. (2014). Update on transparent testa mutants from Arabidopsis thaliana: characterisation of new alleles from an isogenic collection. *Planta* 240(5)**,** 955-970. doi: 10.1007/s00425-014-2088-0.

Jakoby, M.J., Falkenhan, D., Mader, M.T., Brininstool, G., Wischnitzki, E., Platz, N., et al. (2008). Transcriptional profiling of mature Arabidopsis trichomes reveals that NOECK encodes the MIXTA-like transcriptional regulator MYB106. *Plant Physiol* 148(3)**,** 1583-1602. doi: 10.1104/pp.108.126979.

Koornneef, M. (1981). The complex syndrome of ttg mutants. *Arabidopsis Information Service* 18**,** 45-51.

Larkin, J.C., Oppenheimer, D.G., Lloyd, A.M., Paparozzi, E.T., and Marks, M.D. (1994). Roles of the GLABROUS1 and TRANSPARENT TESTA GLABRA Genes in Arabidopsis Trichome Development. *Plant Cell* 6(8)**,** 1065-1076. doi: 10.1105/tpc.6.8.1065.

Larkin, J.C., Walker, J.D., Bolognesi-Winfield, A.C., Gray, J.C., and Walker, A.R. (1999). Allele-specific interactions between ttg and gl1 during trichome development in Arabidopsis thaliana. *Genetics* 151(4)**,** 1591-1604.

McNellis, T.W., von Arnim, A.G., Araki, T., Komeda, Y., Misera, S., and Deng, X.W. (1994). Genetic and molecular analysis of an allelic series of cop1 mutants suggests functional roles for the multiple protein domains. *Plant Cell* 6(4)**,** 487-500. doi: 10.1105/tpc.6.4.487.

Pesch, M., Schultheiss, I., Digiuni, S., Uhrig, J.F., and Hulskamp, M. (2013). Mutual control of intracellular localisation of the patterning proteins AtMYC1, GL1 and TRY/CPC in Arabidopsis. *Development* 140(16)**,** 3456-3467. doi: 10.1242/dev.094698.

Rosso, M.G., Li, Y., Strizhov, N., Reiss, B., Dekker, K., and Weisshaar, B. (2003). An Arabidopsis thaliana T-DNA mutagenized population (GABI-Kat) for flanking sequence tag-based reverse genetics. *Plant Mol Biol* 53(1-2)**,** 247-259. doi: 10.1023/B:PLAN.0000009297.37235.4a.

Walker, A.R., Davison, P.A., Bolognesi-Winfield, A.C., James, C.M., Srinivasan, N., Blundell, T.L., et al. (1999). The TRANSPARENT TESTA GLABRA1 locus, which regulates trichome differentiation and anthocyanin biosynthesis in Arabidopsis, encodes a WD40 repeat protein. *Plant Cell* 11(7)**,** 1337-1350. doi: 10.1105/tpc.11.7.1337.

Wester, K., Digiuni, S., Geier, F., Timmer, J., Fleck, C., and Hulskamp, M. (2009). Functional diversity of R3 single-repeat genes in trichome development. *Development* 136(9)**,** 1487-1496. doi: 10.1242/dev.021733.
