# Supplementary material for: TRANSPARENT TESTA GLABRA 1 participates in flowering time regulation in Arabidopsis thaliana
Source: PeerJ. 2020 Jan 20;8:e8303. doi: 10.7717/peerj.8303 (PMC6977477; doi:10.7717/peerj.8303)
Supplement: Table S8 [file peerj-08-8303-s018.docx]

**Supplementary Table S8.** Primers. AGI codes were extracted from TAIR (www.arabidopsis.org).

| **Name** | **Gene/**  **mutant/**  **insertion** | **AGI** | **Sequence** | **Citation/link for primer** |
| --- | --- | --- | --- | --- |
| **Genotyping** | | | | |
| ANS584 | *ttg1-1^1^* | AT5G24520 | CTGATACGCCTTTGTTAAGAC | (Jaegle et al., 2016) |
| ANS585 | *ttg1-1^1^* | AT5G24520 | CAATACCAATCCAATCAGGCTGCGAAGAAGACCACTGCAGCT | (Jaegle et al., 2016) |
| ANS584 | *ttg1-9^2^* | AT5G24520 | CTGATACGCCTTTGTTAAGAC | (Jaegle et al., 2016) |
| ANS632 ttg1-9 dCAPS-as | *ttg1-9^2^* | AT5G24520 | CCCAAATAAGAGCCTGTGTATCATCACCTCGA | this study |
| ANS635 ttg1-10-s | *ttg1-10^3^* | AT5G24520 | GATTAGAGTGTCACATATTAATGTTG | this study |
| ANS636 ttg1-10 dCAPS-as | *ttg1-10^3^* | AT5G24520 | CTGATTCGATTCGAGATTTGGCTT**G**TT**A**A | this study |
| ANS633 ttg1-11-s | *ttg1-11^4^* | AT5G24520 | CATCCTTATCCTCCAACAAAGCTAATG | this study |
| ANS634 ttg1-11 dCAPS-as | *ttg1-11^4^* | AT5G24520 | ATCGTACACGTCGTATCAATACTACA**G**GT**A**C | this study |
| ANS635 ttg1-10-s | *ttg1-21* | AT5G24520 | GATTAGAGTGTCACATATTAATGTTG | this study |
| ANS536 (GKo8409) | *ttg1-21* | AT5G24520 | ATATTGACCATCATACTCATTGC | www.gabi-kat.de/faq/ vector-a-primer-info.html |
| ANS536 (GKo8409) | *ttg1-22* | AT5G24520 | ATATTGACCATCATACTCATTGC | www.gabi-kat.de/faq/ vector-a-primer-info.html |
| ANS585 | *ttg1-22* | AT5G24520 | CAATACCAATCCAATCAGGCTGCGAAGAAGACCACTGCAGCT | (Jaegle et al., 2016) |
| ANS183 COP1dCAPS cop1-4 | *cop1-4^5^* | AT2G32950 | CCAAAGAAGGATGCGCTGAGTGGGTCAGATACG | (Schrader et al., 2013) |
| ANS184 cop1-4 genotyping as genomic | *cop1-4^5^* | AT2G32950 | TCTCGAGCTGTCAATCCAGATGACCAAG | (Schrader et al., 2013) |
| ANS535 GK545D05 | *gl3-3* | AT5G41315 | ATGGATACTATAATGGAGATATCAA | (Appelhagen et al., 2011) |
| ANS537 gl3-3as | *gl3-3* | AT5G41315 | GTTACATAGCAACTACATCAATAC | this study |
| ANS538 egl3-19114s | *egl3-19117* | AT1G63650 | TAACCGATTCTAGCAAATCTGTTTC | this study |
| ANS539 egl3-19114as | *egl3-19117* | AT1G63650 | GCAATCAACATTAAACATTACATATC | this study |
| ANS523 bHLH12 | *bHLH12/myc1* | AT4G00480 | ATGTCTTTGACAATGGCTGATGGTGTAGAAG | this study |
| ANS545 bHLH12as | *bHLH12/myc1* | AT4G00480 | GACATTTCTCATTACTTCTTTATTG | this study |
| ANS525 Lba1 | SALK |  | TGGTTCACGTAGTGGGCCATCG | http://signal.salk.edu/tdna_FAQs.html |
| ANS536 (GKo8409) | GABI-Kat |  | ATATTGACCATCATACTCATTGC | www.gabi-kat.de/faq/ vector-a-primer-info.html |
| **Test for genomic DNA in cDNA preparations** | | | | |
| ANS167 (EF1a-UP) | *EF1ALPHA* | AT5G60390 | ATGCCCCAGGACATCGTGATTTCAT | (Kirik et al., 2007) |
| ANS168 (EF1a-RP) | *EF1ALPHA* | AT5G60390 | TTGGCGGCACCCTTAGCTGGATCA | (Kirik et al., 2007) |
| **qPCR** |  |  |  |  |
| ANS845 PRR9_qPCR_s | *PRR9* | AT2G46790 | CTGATGCGTCGGCCTTCTC | (Wang et al., 2011) |
| ANS846_PRR9_qPCRas | *PRR9* | AT2G46790 | CGACGGCTTTTTCTGCTGACT | (Wang et al., 2011) |
| ANS847 PRR7_qPCR_s | *PRR7* | AT5G02810 | AGAGGTGCTTCCGAAAGAAGGT | (Wang et al., 2011) |
| ANS848_PRR7_qPCRas | *PRR7* | AT5G02810 | CTGCTAGTTTTTTCCGGCTTTG | (Wang et al., 2011) |
| ANS849 PRR5_qPCR_s* | *PRR5* | AT5G24470 | TTCCGAATGAAGCGAAAGGAC | (Wang et al., 2011) |
| ANS850_PRR5_qPCRas* | *PRR5* | AT5G24470 | CCGGCTCTCGTAACGAACCT | (Wang et al., 2011) |
| ANS853 FLC_qPCRs | *FLC* | AT5G10140 | TGTTCAACTGGAGGAACACCTTG | (Wang et al., 2014) |
| ANS854 FLC_qPCRas | *FLC* | AT5G10140 | AGCTTCAACATGAGTTCGGTCTTC | (Wang et al., 2014) |
| ANS855 GI_qPCRs | *GI* | AT1G22770 | GGGTAAATATGCTGCTGGAGA | (Nakamichi et al., 2007) |
| ANS856 GI_qPCRas | *GI* | AT1G22770 | CAGTATGACACCAGCTCCATT | (Nakamichi et al., 2007) |
| ANS519 (UBQ10 F3) | *UBQ10* | AT4G05320 | GGCCTTGTATAATCCCTGATGAATAAG | (Maier et al., 2013) |
| ANS520 (UBQ10 R3) | *UBQ10* | AT4G05320 | AAAGAGATAACAGGAACGGAAACATAGT | (Maier et al., 2013) |
| ANS788 (TOC1s) | *TOC1* | AT5G61380 | ATCTTCGCAGAATCCCTGTGATA | (Wenden et al., 2011) |
| ANS789 (TOC1as) | *TOC1* | AT5G61380 | GCACCTAGCTTCAAGCACTTTACA | (Wenden et al., 2011) |
| ANS790 (CCA1s) | *CCA1* | AT2G46830 | TCTGTGTCTGACGAGGGTCGAATT | (Shin et al., 2017) |
| ANS791 (CCA1as) | *CCA1* | AT2G46830 | ACTTTGCGGCAATACCTCTCTGG | (Shin et al., 2017) |
| ANS792 (LHYs) | *LHY* | AT1G01060 | CAACAGCAACAACAATGCAACTAC | (Shin et al., 2017) |
| ANS793 (LHYas) | *LHY* | AT1G01060 | AGAGAGCCTGAAACGCTATACGA | (Shin et al., 2017) |
| ANS799 (AP2s) | *AP2* | AT4G36920 | CGAAGCTGCTAGAGCTTACG | (Grigorova et al., 2011) |
| ANS800 (AP2as) | *AP2* | AT4G36920 | CGAGGTTGTGATCTTGTGGAGTAG | (Grigorova et al., 2011) |
| ANS801 (TOE1s) | *TOE1* | AT2G28550 | TGATGAGTAACTGGGGATGGC | (Zhang et al., 2015) |
| ANS802 (TOE1as) | *TOE1* | AT2G28550 | GCATTGTCATTGGGAGGTTG | (Zhang et al., 2015) |
| ANS803 (TOE2s) | *TOE2* | AT5G60120 | CATCATCCTTCTCAGCCCTTC | (Zhang et al., 2015) |
| ANS804 (TOE2as) | *TOE2* | AT5G60120 | GCCTTCCAACTTATTCCAACC | (Zhang et al., 2015) |
| ANS805 (TOE3s) | *TOE3* | AT5G67180 | AAGAATCCGACAGTAGAGGG | (Zou et al., 2013) |
| ANS806 (TOE3as) | *TOE3* | AT5G67180 | CTTACCGACACTATTGAAACCG | (Zou et al., 2013) |
| ANS807 (SMZs) | *SMZ* | AT3G54990 | CGTAGCTCCCAATATCGTGGC | (Zhang et al., 2015) |
| ANS808 (SMZas) | *SMZ* | AT3G54990 | GCGTAAGCAGTATCAAACCCG | (Zhang et al., 2015) |
| ANS809 (SNZs) | *SNZ* | AT2G39250 | CAGCAGATTATTACATGGGTTTG | (Yu et al., 2012) |
| ANS810 (SNZas) | *SNZ* | AT2G39250 | GGTTTAATTTCTGTGATCGGTAGA | (Yu et al., 2012) |
| ANS811 (TEM1) | *TEM1* | AT1G25560 | ACCAGACCGGCAATTGTATATCCAC | (Yu et al., 2012) |
| ANS812 (TEM1as) | *TEM1* | AT1G25560 | ATCTCTCTTGCCAACACACTCTACTG | (Yu et al., 2012) |
| ANS813 (TEM2s) | *TEM2* | AT1G68840 | GACTAGAGCGGCAGTTATATAT | (Yu et al., 2012) |
| ANS814 (TEM2as) | *TEM2* | AT1G68840 | CTTTCCACCGCAAACGGCCA | (Yu et al., 2012) |
| ANS815 (SVPs) | *SVP* | AT2G22540 | CAAGGACTTGACATTGAAGAGCTTCA | (Li et al., 2008) |
| ANS816 (SVPas) | *SVP* | AT2G22540 | CTGATCTCACTCATAATCTTGTCAC | (Li et al., 2008) |
| ANS817 (SOC1s) | *SOC1* | AT2G45660 | AGCTGCAGAAAACGAGAAGCTCTCTG | (Li et al., 2008) |
| ANS818 (SOC1as) | *SOC1* | AT2G45660 | GGGCTACTCTCTTCATCACCTCTTCC | (Li et al., 2008) |
| BP018_COqPCR_s | *CO* | AT5G15840 | ATGCCTTCCTCGAAGCATACC | (Hayama et al., 2017) |
| BP019_COqPCR_as | *CO* | AT5G15840 | GCATGTGTCACAACAGCTTCAC | (Hayama et al., 2017) |
| BP020_FTqPCR_s | *FT* | AT1G65480 | TCAGAGGGAGAGTGGCTG | (Hayama et al., 2017) |
| BP021_FTqPCR_as | *FT* | AT1G65480 | TCACCGTTCGTTACTCGTATC | (Hayama et al., 2017) |
| BP005_TTG1qPCR_s (endo) | *TTG1* | AT5G24520 | CTGATTGGATTGGTATTGCT | this study |
| BP006_TTG1qPCR_as (endo) | *TTG1* | AT5G24520 | CGCAAACCAAACCTACTTAC | this study |
| New_2_fwd_qPCR_AtTTG1 (both) | *TTG1* | AT5G24520 | GTTGCTGAGCTTGAAAGACATC | provided by J. Schiffner |
| BP049_TTG1_no_LWD_fwd | *TTG1* | AT5G24520 | ACTATGCCTGTTGCTGAGCTTG | this study |
| New_2_rev_qPCR_AtTTG1 (both/no LWD) | *TTG1* | AT5G24520 | CTGTGTATCATCACCACCAGAA | provided by J. Schiffner |
| **Cloning** |  |  |  |  |
| ANS821 TOC1_GWs | *TOC1/PRR1* | AT5G61380 | GGGGACAAGTTTGTACAAAAAAGCAGGCTTAATGGATTTGAACGGTGAGTG | this study |
| ANS822 TOC1_Gwas | *TOC1/PRR1* | AT5G61380 | GGGGACCACTTTGTACAAGAAAGCTGGGTTTCAAGTTCCCAAAGCATCAT | this study |
| BP012_PRR5_GW_s | *PRR5* | AT5G24470 | GGGGACAAGTTTGTACAAAAAAGCAGGCTTAATGACTAGTAGCGAGGAAGT | this study |
| BP013_PRR5_GW_as | *PRR5* | AT5G24470 | GGGGACCACTTTGTACAAGAAAGCTGGGTTCTATGGAGCTTGTGTGGATT | this study |
| ANS823PRR7_GWs | *PRR7* | AT5G02810 | GGGGACAAGTTTGTACAAAAAAGCAGGCTTAATGAATGCTAATGAGGAGGG | this study |
| ANS824 PRR7_Gwas | *PRR7* | AT5G02810 | GGGGACCACTTTGTACAAGAAAGCTGGGTTTTAGCTATCCTCAATGTTTT | this study |
| ANS825 PRR9_GWs | *PRR9* | AT2G46790 | GGGGACAAGTTTGTACAAAAAAGCAGGCTTAATGGGGGAGATTGTGGTTTT | this study |
| ANS826 PRR9_GWas | *PRR9* | AT2G46790 | GGGGACCACTTTGTACAAGAAAGCTGGGTTTCATGATTTTGTAGACGCGT | this study |
| ANS393 CFP attB1 | *CFP-attB1* | - | GGGGACAAGTTTGTACAAAAAAGCAGGCTTAATGGTGAGCAAGGGCGAGGA | this study |
| ANS235 RFP-HA-attB1 rev | *CFP-attB1* | - | GGGGACCACTTTGTACAAGAAAGCTGGGTCTCAGAAGCCTGCCTTCTTGTAC | (Schrader et al., 2013) |
| BP016_BHLH92.1_GW_s | *bHLH92* | AT5G43650 | GGGGACAAGTTTGTACAAAAAAGCAGGCTTAATGGATAACTTTTTTCTAGG | this study |
| BP017_BHLH92.1_GW_as | *bHLH92* | AT5G43650 | GGGGACCACTTTGTACAAGAAAGCTGGGTTTTAGTAGTCTTTGTAAAAGG | this study |

^1^PvuII, ^2^XhoI, ^3^HpaI, ^4^KpnI, ^5^SnaBI used for dCAPS primers. (The dCAPS Finder 2.0 was used for dCAPS primer design (Neff et al., 2002).) *The following ratios were used for qPCR: 2:1 (ANS849:ANS850)

Appelhagen, I., Jahns, O., Bartelniewoehner, L., Sagasser, M., Weisshaar, B., and Stracke, R. (2011). Leucoanthocyanidin Dioxygenase in Arabidopsis thaliana: characterization of mutant alleles and regulation by MYB-BHLH-TTG1 transcription factor complexes. *Gene* 484(1-2)**,** 61-68. doi: 10.1016/j.gene.2011.05.031.

Grigorova, B., Mara, C., Hollender, C., Sijacic, P., Chen, X., and Liu, Z. (2011). LEUNIG and SEUSS co-repressors regulate miR172 expression in Arabidopsis flowers. *Development* 138(12)**,** 2451-2456. doi: 10.1242/dev.058362.

Hayama, R., Sarid-Krebs, L., Richter, R., Fernandez, V., Jang, S., and Coupland, G. (2017). PSEUDO RESPONSE REGULATORs stabilize CONSTANS protein to promote flowering in response to day length. *EMBO J* 36(7)**,** 904-918. doi: 10.15252/embj.201693907.

Jaegle, B., Uroic, M.K., Holtkotte, X., Lucas, C., Termath, A.O., Schmalz, H.G., et al. (2016). A fast and simple LC-MS-based characterization of the flavonoid biosynthesis pathway for few seed(ling)s. *BMC Plant Biol* 16(1)**,** 190. doi: 10.1186/s12870-016-0880-7.

Kirik, V., Herrmann, U., Parupalli, C., Sedbrook, J.C., Ehrhardt, D.W., and Hulskamp, M. (2007). CLASP localizes in two discrete patterns on cortical microtubules and is required for cell morphogenesis and cell division in Arabidopsis. *Journal of Cell Science* 120(24)**,** 4416-4425. doi: 10.1242/jcs.024950.

Li, D., Liu, C., Shen, L., Wu, Y., Chen, H., Robertson, M., et al. (2008). A repressor complex governs the integration of flowering signals in Arabidopsis. *Dev Cell* 15(1)**,** 110-120. doi: 10.1016/j.devcel.2008.05.002.

Maier, A., Schrader, A., Kokkelink, L., Falke, C., Welter, B., Iniesto, E., et al. (2013). Light and the E3 ubiquitin ligase COP1/SPA control the protein stability of the MYB transcription factors PAP1 and PAP2 involved in anthocyanin accumulation in Arabidopsis. *Plant J* 74(4)**,** 638-651. doi: 10.1111/tpj.12153.

Nakamichi, N., Kita, M., Niinuma, K., Ito, S., Yamashino, T., Mizoguchi, T., et al. (2007). Arabidopsis clock-associated pseudo-response regulators PRR9, PRR7 and PRR5 coordinately and positively regulate flowering time through the canonical CONSTANS-dependent photoperiodic pathway. *Plant Cell Physiol* 48(6)**,** 822-832. doi: 10.1093/pcp/pcm056.

Neff, M.M., Turk, E., and Kalishman, M. (2002). Web-based primer design for single nucleotide polymorphism analysis. *Trends Genet* 18(12)**,** 613-615.

Schrader, A., Welter, B., Hulskamp, M., Hoecker, U., and Uhrig, J.F. (2013). MIDGET connects COP1-dependent development with endoreduplication in Arabidopsis thaliana. *Plant J* 75(1)**,** 67-79. doi: 10.1111/tpj.12199.

Shin, J., Sanchez-Villarreal, A., Davis, A.M., Du, S.X., Berendzen, K.W., Koncz, C., et al. (2017). The metabolic sensor AKIN10 modulates the Arabidopsis circadian clock in a light-dependent manner. *Plant Cell Environ* 40(7)**,** 997-1008. doi: 10.1111/pce.12903.

Wang, C.Q., Guthrie, C., Sarmast, M.K., and Dehesh, K. (2014). BBX19 interacts with CONSTANS to repress FLOWERING LOCUS T transcription, defining a flowering time checkpoint in Arabidopsis. *Plant Cell* 26(9)**,** 3589-3602. doi: 10.1105/tpc.114.130252.

Wang, Y., Wu, J.F., Nakamichi, N., Sakakibara, H., Nam, H.G., and Wu, S.H. (2011). LIGHT-REGULATED WD1 and PSEUDO-RESPONSE REGULATOR9 form a positive feedback regulatory loop in the Arabidopsis circadian clock. *Plant Cell* 23(2)**,** 486-498. doi: 10.1105/tpc.110.081661.

Wenden, B., Kozma-Bognar, L., Edwards, K.D., Hall, A.J., Locke, J.C., and Millar, A.J. (2011). Light inputs shape the Arabidopsis circadian system. *Plant J* 66(3)**,** 480-491. doi: 10.1111/j.1365-313X.2011.04505.x.

Yu, S., Galvao, V.C., Zhang, Y.C., Horrer, D., Zhang, T.Q., Hao, Y.H., et al. (2012). Gibberellin regulates the Arabidopsis floral transition through miR156-targeted SQUAMOSA promoter binding-like transcription factors. *Plant Cell* 24(8)**,** 3320-3332. doi: 10.1105/tpc.112.101014.

Zhang, B., Wang, L., Zeng, L., Zhang, C., and Ma, H. (2015). Arabidopsis TOE proteins convey a photoperiodic signal to antagonize CONSTANS and regulate flowering time. *Genes Dev* 29(9)**,** 975-987. doi: 10.1101/gad.251520.114.

Zou, Y., Wang, Y., Wang, L., Yang, L., Wang, R., and Li, X. (2013). miR172b controls the transition to autotrophic development inhibited by ABA in Arabidopsis. *PLoS One* 8(5)**,** e64770. doi: 10.1371/journal.pone.0064770.
